# Amyloid Precursor Proteins Are Dynamically Trafficked and Processed during Neuronal Development

**DOI:** 10.3389/fnmol.2016.00130

**Published:** 2016-11-25

**Authors:** Jenna M. Ramaker, Robert S. Cargill, Tracy L. Swanson, Hanil Quirindongo, Marlène Cassar, Doris Kretzschmar, Philip F. Copenhaver

**Affiliations:** ^1^Department of Cell, Developmental and Cancer Biology, Oregon Health and Science UniversityPortland, OR, USA; ^2^Neuroscience Graduate Program, Oregon Health and Science UniversityPortland, OR, USA; ^3^Oregon Institute of Occupational Health Sciences, Oregon Health and Science UniversityPortland, OR, USA

**Keywords:** APPL, *M. sexta*, *D. melanogaster*, secretase, migration, outgrowth, transport, amphisome

## Abstract

Proteolytic processing of the Amyloid Precursor Protein (APP) produces beta-amyloid (Aβ) peptide fragments that accumulate in Alzheimer's Disease (AD), but APP may also regulate multiple aspects of neuronal development, albeit via mechanisms that are not well understood. APP is a member of a family of transmembrane glycoproteins expressed by all higher organisms, including two mammalian orthologs (APLP1 and APLP2) that have complicated investigations into the specific activities of APP. By comparison, insects express only a single APP-related protein (APP-Like, or APPL) that contains the same protein interaction domains identified in APP. However, unlike its mammalian orthologs, APPL is only expressed by neurons, greatly simplifying an analysis of its functions *in vivo*. Like APP, APPL is processed by secretases to generate a similar array of extracellular and intracellular cleavage fragments, as well as an Aβ-like fragment that can induce neurotoxic responses in the brain. Exploiting the complementary advantages of two insect models (*Drosophila melanogaster* and *Manduca sexta*), we have investigated the regulation of APPL trafficking and processing with respect to different aspects of neuronal development. By comparing the behavior of endogenously expressed APPL with fluorescently tagged versions of APPL and APP, we have shown that some full-length protein is consistently trafficked into the most motile regions of developing neurons both *in vitro* and *in vivo.* Concurrently, much of the holoprotein is rapidly processed into N- and C-terminal fragments that undergo bi-directional transport within distinct vesicle populations. Unexpectedly, we also discovered that APPL can be transiently sequestered into an amphisome-like compartment in developing neurons, while manipulations targeting APPL cleavage altered their motile behavior in cultured embryos. These data suggest that multiple mechanisms restrict the bioavailability of the holoprotein to regulate APPL-dependent responses within the nervous system. Lastly, targeted expression of our double-tagged constructs (combined with time-lapse imaging) revealed that APP family proteins are subject to complex patterns of trafficking and processing that vary dramatically between different neuronal subtypes. In combination, our results provide a new perspective on how the regulation of APP family proteins can be modulated to accommodate a variety of cell type-specific responses within the embryonic and adult nervous system.

## Introduction

The Amyloid Precursor Protein (APP) is the source of beta-amyloid (Aβ) peptide fragments that accumulate in Alzheimer's disease (AD), but APP also has been implicated in multiple aspects of neurogenesis and neuronal differentiation (Jung and Herms, 2012; Sosa et al., 2013; Nicolas and Hassan, 2014). APP is a member of an evolutionarily ancient family of type-1 transmembrane glycoproteins found in all higher organisms, typified by highly conserved extracellular and intracellular protein interaction motifs that permit transmembrane signaling (De Strooper and Annaert, 2000; Turner et al., 2003; van der Kant and Goldstein, 2015). Numerous studies have demonstrated that APP can function as a neuronal receptor, capable of binding a variety of candidate ligands and transducing intracellular responses that modulate cell adhesion, neuronal outgrowth, and migration (Osterfield et al., 2008; Nikolaev et al., 2009; Rama et al., 2012; Rice et al., 2013). In support of this model, APP can be detected in the growing processes and focal adhesion complexes of cultured cells, suggesting that APP signaling might regulate the cytoskeletal dynamics required for neuronal outgrowth and migration (Sabo et al., 2003; Wang et al., 2005; Young-Pearse et al., 2007; Ramaker et al., 2013). In particular, members of the APP family can function as unconventional G protein-coupled receptors (Nishimoto et al., 1993; Giambarella et al., 1997; Swanson et al., 2005), transducing responses to local cues via the heterotrimeric G protein Goα to regulate neuronal guidance (Brouillet et al., 1999; Ramaker et al., 2013, 2016). Conversely, APP-Goα interactions have been found to decrease in patients suffering from AD, suggesting that the dysregulation of normal APP-Goα signaling might provoke neuropathological responses (Shaked et al., 2009; Sola Vigo et al., 2009; Milosch et al., 2014).

**Figure 1:**
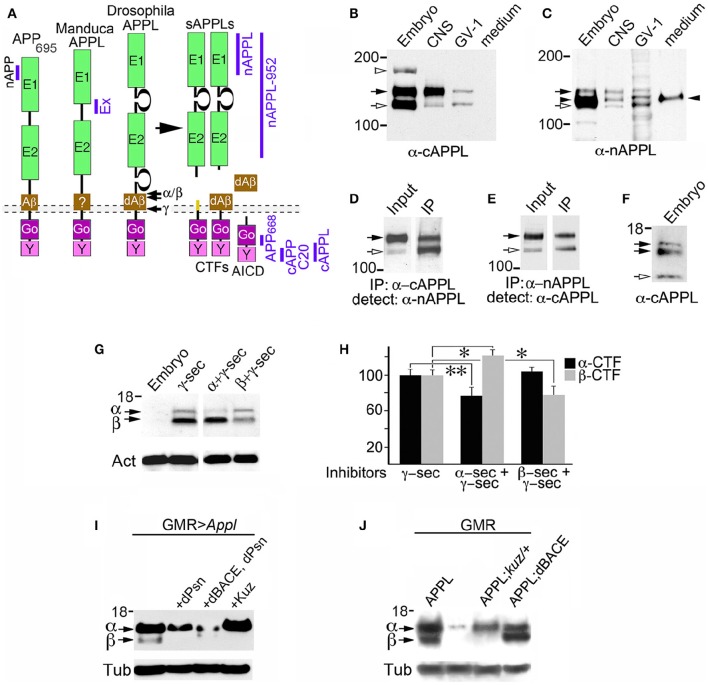
**Insect APPL is cleaved by the same secretase classes that process APP**. **(A)** Schematic image of the primary domains shared by human APP_695_ and APPL in *Drosophila* and *Manduca*. All APP family members contain similar extracellular domains (E1 and E2) that can interact with potential binding partners; a highly conserved cytoplasmic domain (Go) that directly interacts with the heterotrimeric G protein Goα; and a C-terminal tyrosine-based sorting motif (Y) that interacts with a variety of intracellular adapter and signaling molecules. *Drosophila* APPL contains larger non-conserved regions on either side of the E2 domain that increase the overall size of the holoprotein, and an Aβ-like domain (dAβ) with neurotoxic activity when cleaved from the holoprotein; the biological activity of this domain in *Manduca* APPL has not yet been verified. Similar to the cleavage products of APP_695_, processing of insect APPL by α- and β-secretases produces soluble ectodomain fragments (sAPPLs) and short transmembrane C-terminal fragments (CTFs); subsequent cleavage of the CTFs by γ-secretase produces an APPL intracellular domain (AICD), as well the dAβ peptide or a p3-like fragment (not shown). Labeled blue bars indicate the epitopes recognized by antibodies against APPL or APP that were used in this study (as described in the Materials and Methods Section). **(B,C)** Western blots of lysates prepared from *Manduca* embryos (65 HPF), 5th instar CNS, *Manduca* GV-1 cells (which endogenously express APPL), and concentrated medium harvested from the GV-1 cultures. **(B)** Immunoblotting with anti-cAPPL detects both the mature (black arrow) and immature (open arrow) full-length forms of APPL in all three lysates but not in GV-1 cell medium; a larger band (~165 kDa; open arrowhead) detected in mid-stage embryos might represent an additional post-translational modification that is developmentally regulated (as previously reported; Swanson et al., 2005). **(C)** Immunoblotting with anti-nAPPL detects the same mature (black arrow) and immature (open arrow) full-length forms of APPL, plus cleaved ectodomain fragments (sAPPLs) that are also present in GV-1 medium (black arrowhead). The relative intensity of this ectodomain band reflects the rapid processing of full-length APPL; sAPPL produced by α- vs. β-secretases were not distinguished in this blot. **(D,E)** Cross-immunoprecipitation of *Manduca* embryonic lysates with N- and C-terminal-specific antibodies against APPL. **(D)** Embryonic lysate (input) that was immunoprecipitated with anti-cAPPL (IP) and immunoblotted with anti-nAPPL. **(E)** Embryonic lysate (input) that was immunoprecipitated with anti-nAPPL (IP) and immunoblotted with anti-cAPPL; both antibodies recognize mature (black arrow) and immature (open arrow) forms of full-length APPL. **(F)** Western blot of *Manduca* embryo lysate (lower portion) labeled with anti-cAPPL reveals two CTFs (black arrows) and a candidate AICD fragment (open arrowhead). **(G)** Western blot of *Manduca* embryo lysates treated with different secretase inhibitors; in this shorter exposure (compared to **F**), neither CTF was detected (black arrows). In lysates of embryos treated with a γ–secretase inhibitor (lane 2), both CTFs were readily detected. Treatment with a combination of α- plus γ-secretase inhibitors reduced the relative abundance of the upper CTF band, whereas treatment with β- plus γ-secretase inhibitors reduced the lower CTF band. Separate band labeled with “Act” indicates anti-actin (~42 kDa) as a loading control. **(H)** Quantification of CTF abundance in western blots of embryonic lysates (as illustrated in **G**). Treatment with α- plus γ-secretase inhibitors caused a significant decrease in α-CTF levels (^**^p = 0.0002) and a more moderate increase in β-CTF levels (^*^p = 0.041). Treatment with β- plus γ-secretase inhibitors caused a significant reduction in β-CTF (^*^p = 0.041) but did not affect α-CTF levels (*p* = 0.101). Relative intensities were normalized against γ-secretase-treated lysates in each immunoblot. *N* ≥ 10 for each group; histograms show means ± SEM. Statistical comparisons were performed using one-way ANOVA followed by pairwise Student's two-tailed *t*-tests with the Bonferroni correction to obtain reported *p*-values. **(I)** Western blots of head lysates from flies expressing additional APPL in the eye (GMR-GAL4; UAS-Appl), immunoblotted with anti-cAPPL. Lane 1, both α- and β-CTFs (arrows) could be readily detected in GMR>Appl flies. Lane 2, expressing additional *Drosophila* Presenilin in this line (via UAS-dPsn) reduced α- and β-CTFs (β-CTF was no longer detectable at this exposure). Lane 3, expressing additional dPsn plus *Drosophila* BACE (via UAS-dPsn + UAS-dBACE) preferentially reduced α-CTF levels (β-CTF was still detectable, compared to lane 2). Lane 4, expressing additional Kuzbanian in this line (via UAS-Kuz) caused a marked increase in α-CTF and a corresponding reduction in β-CTF levels. **(J)** Western blots of head lysates from flies carrying the eye-specific promoter construct GAL4-GMR, immunoblotted with anti-cAPPL. Lane 1, in flies overexpressing APPL (via UAS-Appl), both α- and β-CTFs (arrows) could be readily detected (as in panel I, lane 1). Lane 2, in GMR-GAL4 control flies, only the α-CTF band was faintly detected at this exposure. Lane 3, both CTFs were reduced in flies lacking one copy of the α-secretase Kuzbanian (*kuz/*+). Lane 4, co-expressing additional APPL and dBACE caused a preferential increase in β-CTF levels. Separate bands in **(I,J)** labeled with “Tub” show anti-tubulin (~55 kDa) as a loading control.

In addition to its potential role as a transmembrane receptor, numerous functions have been ascribed to the cleavage products of APP that are generated via proteolytic processing of the holoprotein by membrane-associated secretases (Figure 1). In the amyloidogenic pathway, APP is initially cleaved by β-secretase (BACE1) to generate a large soluble ectodomain fragment (sAPPβ) and a shorter, membrane-bound C-terminal fragment (β-CTF), which in turn is cleaved by γ-secretase complexes to produce a small APP intracellular fragment (AICD) and Aβ peptides (Gralle and Ferreira, 2007; Guo et al., 2012). Alternatively, in the non-amyloidogenic pathway, APP is initially cleaved by an α-secretase (typically ADAM10) to generate sAPPα and α-CTF fragments, the latter being further processed by γ-secretase to produce an identical AICD and a p3 peptide that is rapidly degraded (Turner et al., 2003; Haass et al., 2012). A plethora of biological activities have been postulated for many of these fragments, ranging from transcriptional regulation to synaptic remodeling and neurodegenerative responses (Kimberly et al., 2005; Kogel et al., 2012; Zhang et al., 2012; Nhan et al., 2015). Nevertheless, authentic functions for particular APP cleavage products within the developing nervous system remain under debate.

Most models of APP-dependent signaling are based on evidence that the full-length holoprotein can be transported to peripheral regions of neurons before insertion into the plasma membrane, whereupon it undergoes rapid internalization and/or cleavage (e.g., Schubert et al., 1991; Rice et al., 2012; Octave et al., 2013; Sosa et al., 2013; Olsen et al., 2014). In support of this model, rapid anterograde axonal transport of APP has been clearly demonstrated in cell culture (Koo et al., 1990; Seamster et al., 2012; Szpankowski et al., 2012). In contrast, several recent reports have shown that much of the holoprotein is actually processed into different fragments that are assorted to distinct transport vesicles before they exit the cell body (Muresan et al., 2009; Villegas et al., 2014). Although initial studies argued that axonally targeted APP must undergo retrograde transport into the somatodendritic compartment before being processed (Simons et al., 1995; Yamazaki et al., 1995; Kins et al., 2006; Back et al., 2007), more recent work using stem cell-derived neurons suggests the opposite pattern: namely, that a substantial portion of full-length APP is initially inserted into the somatodendritic compartment before being transcytosed and processed in endosomal compartments. Only subsequently are different fragments packaged into distinct vesicle populations for axonal transport (Muresan et al., 2009; Muresan and Ladescu Muresan, 2015). However, because most of these studies were conducted using neuroblastoma cell lines or relied solely on the overexpression of exogenous APP, the significance of these different sorting and processing scenarios with respect to APP-dependent functions in the nervous system remained unclear.

Investigations into the normal roles of APP in mammalian systems have been complicated by the discovery of two closely related family members (APP-Like Protein 1 and 2; or APLP1 and APLP2) that share partially overlapping activities (Turner et al., 2003; Shariati and De Strooper, 2013). In contrast, insects express a single APP ortholog (APP-like, or APPL) that shares all of the canonical features of human APP_695_ (the predominant neuronal isoform), including highly conserved extracellular and intracellular domains that interact with similar classes of ligands and signaling proteins (Luo et al., 1990; Torroja et al., 1999; Ashley et al., 2005; Swanson et al., 2005). Like APP, APPL is subject to proteolytic processing by α, β, and γ-secretases that generate an analogous spectrum of cleavage fragments, including sAPPs, CTFs, AICDs, and an Aβ-like peptide (Luo et al., 1995; Greeve et al., 2004; Carmine-Simmen et al., 2009; Poeck et al., 2012). Also like mammalian APP, APPL plays important roles in the developing nervous system, participating in the control of neuronal migration and synaptic plasticity (Torroja et al., 1999; Ashley et al., 2005; Ramaker et al., 2013; Soldano et al., 2013; Bourdet et al., 2015). Notably, studies in *Drosophila* have shown that defects caused by the loss of APPL can be rescued by the expression of human APP_695_ (Luo et al., 1992; Wentzell et al., 2012), indicating that these proteins are both structurally and functionally homologous. However, unlike mammalian APP (which is expressed by many cell types), APPL is exclusively expressed in neurons, greatly simplifying an analysis of its biological functions *in vivo*. Accordingly, we have exploited the complementary strengths of two different insect models (*Manduca* and *Drosophila*) to investigate the developmental regulation of APPL trafficking and processing within both the developing and adult nervous system. Using a combination of *in vitro* and *in vivo* culture preparations, we have also examined how dynamic changes in the distribution of APPL and its cleavage products relate to the motile behavior of developing neurons, and whether altering APPL processing affects neuronal migratory responses within the nervous system.

## Materials and methods

### Western blotting and cross-immunoprecipitation of *Manduca* and *Drosophila* lysates

These studies were conducted using insect model systems that are exempt from animal research protocols. Synchronous groups of embryos of both sexes were obtained from an in-house colony of *Manduca sexta* and staged using published markers (Copenhaver and Taghert, 1989a,b). Embryos reared at 25°C complete their development in 100 h, so that 1 h post-fertilization (HPF) is equivalent to 1% of development. Staged *Manduca* embryos (50 per group; dissected at 65 HPF) were collected on dry ice, homogenized in 1% Triton lysis buffer (1% Triton X-100, 150 mM NaCl, 50 mM Tris, pH 8) or 1% NP40 lysis buffer (150 mM NaCl, 50 mM Tris, pH 8), and the lysates were clarified by centrifugation at 16,000 rpm for 10 min (Swanson et al., 2005). Soluble proteins were then separated on 10% or 4–12% Criterion polyacrylamide gels (Bio-Rad), transferred to nitrocellulose, and immunoblotted with antibodies diluted in Tris-buffered saline plus 0.1% Tween-20 (Polysorbate) and 5% Carnation dry milk. The immunoblots were then incubated overnight at 4°C with anti-nAPPL (1:5000) or anti-cAPPL (1:2500), diluted in Tris-buffered saline plus 0.1% Tween-20 (Polysorbate) and 5% dry milk. Secondary antibodies coupled to Horseradish Peroxidase (HRP; from Jackson ImmunoResearch) were then applied to the blots at 1:10 K and detected using standard chemiluminescent protocols (with either West Pico or West Femto substrates; Thermo Fisher). To detect CTF fragments in fly lysates, 15 heads per genotype were homogenized in sample buffer. The lysates were then loaded on 4–12% gradient gels and analyzed with our published methods (Tschape et al., 2002). For labeling tagged APP and APPL from transgenic *Drosophila* lines, western blots were stained with anti-GFP (Santa Cruz Biotechnologies SC-8334, 1:1000); anti-DsRed (Clontech # 632393; 1:100); and anti-human APP (clone 22C11; 1:100). Antibodies against tubulin (Developmental Studies Hybridoma Bank #E7; deposited by Michael Klymkowsky) and actin (Sigma Aldrich # A2228) were used to label these proteins as loading controls.

For a cross-immunoprecipitation analysis of endogenous APPL in *Manduca*, supernatants of lysed embryos were prepared as described above. After centrifugation, the supernatants were transferred to Eppendorf tubes, pre-cleared with Protein A/G beads (Santa Cruz Biotechnology), and incubated with either anti-cAPPL or anti-nAPPL for 1–3 h at room temperature. The samples were then incubated with pre-washed beads for 1 h, and the bead-bound antibody complexes were pelleted by centrifugation. After washing in chilled lysis buffer, immunoprecipitated proteins were eluted by boiling in SDS sample buffer for 1 min. The samples were then separated on 10% Criterion polyacrylamide gels, transferred to nitrocellulose, and immunoblotted with the complementary anti-APPL antibody: samples immunoprecipitated with antibodies against the N-terminal domain of APPL were immunoblotted with anti-cAPPL; samples immunoprecipitated antibodies against the C-terminal domain were immunoblotted with anti-nAPPL. In some experiments, we also used rabbit-anti-cAPP (Sigma-Aldrich #A8717) and a custom rabbit-anti-APP antibody (C20), generated against a conserved epitope within the cytoplasmic domain of APP (Hare, 2001); both antibodies recognized the same APPL fragments detected by our cAPPL antibody. As an additional control, we used rabbit anti-nAPPL-EX, generated against the sequence EDDDYTDADDSAWPRPES within *Manduca* APPL (Swanson et al., 2005). Antibody detection was performed as described above.

### Whole-mount immunolabeling of staged *Manduca* embryos

Staged embryos were dissected in defined saline (140 mM NaCl, 5 mM KCl, 28 mM glucose, 40 mM CaCl_2_, and 5 mM HEPES, pH 7.4) to expose the enteric nervous system (ENS), as previously described (Horgan et al., 1995; Ramaker et al., 2013). Alternatively, the embryonic gut was removed to expose the developing central nervous system (CNS) before subsequent processing (Swanson et al., 2005). Preparations were fixed in 4% paraformaldehyde (PFA; Sigma/Aldrich) in phosphate-buffered saline (PBS) for 1 h at room temperature, rinsed in PBS plus 0.1% Triton X-100 (PBST), and incubated for 1 h in blocking solution (10% normal horse serum plus 0.1% sodium azide in PBST). For some antibodies, we also fixed embryos for 1 h in either Bouin's fixative (formalin/aqueous saturated picric acid, 3:1; plus 5% glacial acetic acid); Zamboni's fixative (2% PFA plus 15% aqueous saturated picric acid in sodium phosphate buffer; pH 7.3); or Glyo-Fixx (Thermo-Fisher).

Fixed embryos were then incubated in antibodies diluted in blocking solution for either 1 h at room temperature or overnight at 4°C. The following primary antibodies against APPL and APP were used in this analysis: chicken anti-cAPPL (1:2500), generated against the sequence YENPTYKYFEVKE within the cytoplasmic domain of *Manduca* APPL (Swanson et al., 2005); rabbit anti-nAPPL (#21506; 1:5000), generated against a fusion protein derived from the E1 region of *Manduca* APPL (AA 1–197; Ramaker et al., 2013); and rabbit-anti-*Drosophila* APPL (antiserum 952; gift from Dr. Vivian Budnik), generated against the ectodomain of *Drosophila* APPL (Torroja et al., 1996, 1999). The specificity of these antibodies for APPL has previously been validated (Swanson et al., 2005). In some experiments, we also used the following antibodies against the C-terminal domain shared by APPL and APP: anti-cAPP (Sigma-Aldrich A8717), targeting amino acids 676–695 of human APP_695_; and anti-APP_668_ (Sigma-Aldrich #SAB4300464), targeting AA 666–670 of human APP_695_. Both antibodies specifically recognize *Manduca* APPL, as previously reported (Ramaker et al., 2013).

Embryos were counterstained with either anti-pan Fasciclin II (Fas II; C3 mouse monoclonal; 1:20,000); anti-TM Fas II (1:5000; which is specific for the transmembrane isoform of *Manduca* Fas II); or anti-GPI-Fas II (1:5000; specific for the glycosyl phosphatidylinositol-linked form of Fas II; Wright et al., 1999; Wright and Copenhaver, 2000). Primary antibodies were detected with secondary antibodies conjugated to Alexa Fluor 488, 568, or 647 (Molecular Probes/Life Technologies; 1:1000) or conjugated to Cy3 and DyLight 549 (Jackson ImmunoResearch; at 1:400), diluted in blocking solution. Whole-mount immunolabeled preparations were mounted in Elvanol (Banker and Goslin, 1998) and imaged with an Olympus FluoView 300 laser scanning confocal head mounted on an Olympus BX51 microscope (located in the Oregon Institute of Occupational Health Sciences), or with an inverted Zeiss LSM710 confocal microscope (located in the Advanced Light Microscopy Center of the Jungers Institute, OHSU). Maximum intensity projections of flattened z-stack confocal images were generated using MetaMorph and Fiji software.

### Culture and immunolabeling of *Manduca* neurons and GV1 cells

Primary cultures of *Manduca* neurons were prepared following the methods of Hayashi et al. (Hayashi and Hildebrand, 1990; Hayashi and Levine, 1992). Briefly, ganglia from *Manduca* pupae (P2–P4 stages) were enzymatically dissociated, and suspensions of the dispersed neurons were plated in L-15-based culture medium plus 10% fetal bovine serum (FBS) onto coverslips (pre-coated with a mixture of Concanavalin A and laminin) that had been affixed below 8 mm holes drilled into 35 mm plastic dishes. After 1–14 days, neurons were fixed with 4% PFA, permeabilized with PBST for 10 min, and immunolabeled with anti-cAPPL and anti-nAPPL antibodies. *Manduca* GV1 cells are an ectoderm-derived cell line (Lan et al., 1999; Hiruma and Riddiford, 2004) that endogenously expresses APPL and other neuronal proteins (Lan et al., 1999; Hiruma and Riddiford, 2004; Swanson et al., 2005; Coate et al., 2009). GV1 cells were grown on Lab-Tek tissue culture chamber slides (Nunc #177445) in Grace's complete medium plus 10% FBS to 50% confluence, then fixed and immunolabeled, as previously reported (Coate et al., 2009). Replicate cultures were co-immunolabeled with anti-cAPPL plus each of the following antibodies against cytoplasmic compartment markers, based on published evidence and epitope predictions that these antibodies recognize homologous proteins in *Drosophila* cells: rabbit-anti-Rab4 (Cell Signaling #2167, 1:500; and Abcam #ab87802, 1:200); rabbit-anti-*Drosophila* Rab5 (Abcam #ab31261, 1:500; Wang et al., 2014); rabbit-anti-Rab7 (Abcam #ab77993, 1:500) and rabbit-anti-dRab7 (1:1000; gift of Dr. Patrick Dolph; 1:1000; Chinchore et al., 2009; Wang et al., 2014); rabbit-anti-Rab9 (Abcam #ab179815, 1:200); rabbit-anti-Rab10 (Abcam #ab113947, 1:500); goat-anti-Rab10 (Santa Cruz #sc-6564, 1:200); rabbit-anti-Rab11a (1:1000; gift of Dr. Don Ready; Satoh et al., 2005); mouse-anti-Rab11 (BD Transduction # 610657, 1:200; Wang et al., 2014); Rabbit-anti-*Drosophila* LAMP1 (Abcam #ab30687, 1:500); rabbit-anti-*Drosophila* Lava Lamp (gift of Dr. John Sisson, 1:250; Papoulas et al., 2005); rabbit anti-*Drosophila* VPS4 (rabbit anti-*Drosophila* dVPS4, 1:100; gift of Dr. Harald Stenmark; Rodahl et al., 2009); and rabbit-anti-Evi (Evenless Interrupted, 1:400; gift of Dr. Vivian Budnik; Koles et al., 2012). GV1 cells were also labeled with MitoTracker Green FM and LysoTracker Red DND-99 (Life Technologies), following the manufacturer's protocols. Images of immunolabeled primary neurons and GV1 cells were obtained as described above.

### Secretase inhibitor assays of APPL processing in *Manduca*

Staged *Manduca* embryos (~62–63 HPF) were dissected dorsally to expose the migratory EP cells within the developing ENS, as previously described (Coate et al., 2008; Ramaker et al., 2013). Embryos were then transferred to Eppendorf tubes containing 100 μl defined culture saline (140 mM NaCl, 5 mM KCl, 28 mM glucose, 5 mM HEPES, 4 mM CaCl_2_, pH 7.4; Horgan and Copenhaver, 1998), plus 5–50 μM of each inhibitor or vehicle control solutions. The following secretase inhibitors were used in this analysis, based on published evidence that they inhibit analogous secretases in *Drosophila* (Sinha et al., 1999; Greeve et al., 2004; Groth et al., 2010): α-secretase inhibitors GM6001 (Calbiochem) and GI 254023X (Tocris); β-secretase inhibitors #171601 (Calbiochem) and #565788 (Calbiochem); and γ-secretase inhibitor #565770 (DAPT). After incubation in a 27°C heat block for 2.5 h, the tissue was homogenized in 5 μl pre-warmed lysis buffer containing protease and phosphatase inhibitors (4% SDS, 40% glycerol, 200 mM Tris-HCl, pH 6.7; 99°C). Samples were subsequently centrifuged for 10 min at 16 K rpm to clarify the lysates. The supernatants were separated on Bio-Rad 16.5% Tris-Tricine Criterion gels, and labeled in western blots with anti-cAPPL antibodies, as described above.

### *Manduca* embryonic culture and migration assays

Staged *Manduca* embryos were isolated at ~57 HPF, transferred to Sylgard chambers containing defined saline, and opened dorsally to expose the developing ENS. The EP cells were then directly treated with secretase inhibitors or vehicle control solutions. The preparations were allowed to develop for an additional 5 h at 28°C, then fixed and triple-immunolabeled with anti-nAPPL, anti-cAPPL, and anti-Fas II antibodies (as described above), using Alexa-Fluor conjugated secondary antibodies for detection. To quantify the relative levels of membrane-associated full-length APPL and Fas II (as an independent control), 1.5-μm *z*-stack images (consisting of three sequential 0.5 μm confocal sections) were taken from the membrane regions of leading EP cells that had migrated onto the mid-dorsal band pathways. Three separate regions were imaged in each preparation. The z-stacks were then compressed, and fluorescent intensities within boxed regions of interest (ROI) (spanning the plasma membrane of non-overlapping neurons) were quantified independently for each channel using Fiji/ImageJ software. Background fluorescence levels were determined from equivalent *z*-stack images of adjacent interband muscle regions (devoid of APPL and Fas II expression). The ratios of EP cell-associated immunofluorescence vs. background levels were then used to normalize the relative intensities of each fluorochrome associated with the neuronal membranes. These values were then used to compare relative levels of APPL and Fas II expression between groups. All measurements were performed under linear parameters. To analyze migration and axon outgrowth distances in the culture preparations, the preparations were re-immunolabeled with anti-Fas II (C3), followed by immunodetection with the ABC method (Vector Laboratories), using 3,3′-diaminobenzidine tetrahydrochloride (DAB) reacted with H_2_O_2_ to label the neurons. As previously described, this method produces unambiguous labeling of the neurons and their processes in the developing ENS (Copenhaver and Taghert, 1989b; Wright et al., 1999). The extent of EP cell migration and outgrowth was then analyzed and quantified from *camera lucida* images of the preparations (Ramaker et al., 2013).

### *Drosophila* stocks and UAS lines

To create fluorescently double-tagged constructs of APP family proteins, we used cDNAs encoding APP_695_ (kindly provided by R. Reifegerste, University Hamburg) and *Drosophila* APPL (GH04413, obtained from Research Genetics, Huntsville), both of which were cloned into the pUAST vector (Brand and Perrimon, 1993). Plasmids containing the coding domains for enhanced Green Fluorescent Protein (EGFP) and monomeric red fluorescent protein (mRFP; Clontech) were used to amplify the sequences for the two fluorescent tags; mRFP was then cloned in frame with the 3′ ends of both the APP_695_ and APPL coding domains. To insert signal sequences upstream of the 5′ end of the GFP sequence, we used overlapping primers against the signal sequence from APP_695_ or APPL in sequential PCR reactions. The resulting constructs (containing the appropriate signal sequence plus EGFP) were then cloned 5′ to the APP_695_ and APPL coding domains, from which the original signal sequences had been deleted. The constructs were inserted into the fly genome by standard P-element transformation (Spradling and Rubin, 1982). GMR-GAL4, Ddc-GAL4, ChAT-GAL4, Tdc1-GAL4, elav-GAL4, and UAS-kuz were provided by the Bloomington Stock Center; Appl-GAL4 was provided by Dr. Laura Torroja (Universidad Autónoma de Madrid), and the *Appl*^*d*^ fly line was provided by Dr. Kalpana White (Brandeis University). UAS-dBACE and UAS-APPL have been previously described (Carmine-Simmen et al., 2009). Stocks were maintained and raised under standard conditions.

### *Drosophila* primary neuronal cultures

Primary neuronal cell cultures from *Drosophila* white pupae expressing double-tagged APP or APPL (under the control of the different GAL4 drivers) were prepared as described by Kraft et al. (1998). Briefly, neurons from prepupal brains were dissociated via digestion with a collagenase/dispase solution and triturated through a fire-polished glass pipette. Suspensions of the dispersed neurons were plated in Schneider's Insect Medium plus 10% FBS and 50 μg/ml insulin onto coverslips affixed to drilled 35 mm plastic dishes (as described above). The cultures were maintained for 1–8 days, and then either imaged live on an inverted microscope or fixed and immunolabeled (Wentzell et al., 2014). Primary neuronal cultures were fixed in 4% PFA for 5 min and immunolabeled following the protocol described by Buchner et al. (in Ashburner, 1989). To enhance the detection of fluorescently tagged APP_695_ and APPL in fixed preparations, the neurons were double-stained with rabbit anti-GFP (Santa Cruz Biotechnologies SC-8334, 1:1000) and mouse anti-DsRed (Clontech # 632393; 1:500), followed with Cy2- and Cy3-conjugated secondary antibodies (at a 1:1000 dilution; Jackson ImmunoResearch). Preparations were then mounted in Elvanol and imaged using an Olympus FluoView confocal 300 microscope.

For live-cell imaging experiments, measurements of fluorescent vesicle movements were performed on cultured neurons expressing double-tagged APP_695_ or APPL using a Leica DM-IRBE microscope, equipped with a 63x Plan Apo 1.32 NA objective lens and a MicroMax Interline CCD camera (Princeton Instruments). Images were taken every 2 s for 98 s, and analyzed using the kymograph function in MetaMorph (Molecular Devices). For each movie frame, the brightest pixel within a 2 μm corridor along the axis of a neurite was displayed at the corresponding location on a kymograph. The fluorescence patterns for all 50 movie frames were then displayed graphically as an adjacent series, whereby the x-axis of each graph represented time and the y-axis represented distance along the process.

### Immunolabeling and live imaging of *Drosophila* brains

Live imaging of intact 3rd instar larvae expressing double-tagged APP/APPL proteins in neurons was performed by immobilizing the larvae in gelatin/glycerol mounting medium (Ashburner, 1989), and images were collected with a Zeiss Axioscope 2 microscope. To detect expression within the fly CNS, adult brains were dissected as unfixed whole-mount preparations in *Drosophila* Ringer's solution and imaged by confocal microscopy. To image the transport dynamics of vesicles within intact tissues, brain-eye disc complexes from third instar larvae or brains from adult flies were dissected and transferred to a small chamber containing Ringer's solution. Imaging was performed using a Yokogawa CSU-10 spinning disc confocal head mounted on a Nikon TE2000 inverted microscope, equipped with 60x/1.45 NA Plan Apo objective and illuminated with an Innova 70C Spectrum ion laser. Dual-color recordings were acquired every 2 s by fast sequential imaging (500 ms exposure per channel), captured with an Orca ER CCD camera (Hamamatsu Instruments).

### Statistical methods

Relative levels of α- and β-CTF fragments were quantified from western blots (Figures 1G,H), using our published methods (Ramaker et al., 2013). Briefly, immunoblots were visualized by chemiluminescence, and scanned TIFF images of the blots were used to quantify relative pixel intensities within ROI using Fiji. The values within each lane were then normalized to intensity values for actin (for Figure 1G) and tubulin (for Figures 1I,J), used as loading controls. One-way Analysis of Variance (ANOVA) was performed to determine if mean values differed significantly among any of the groups, followed by pairwise Student's two-tailed *t*-tests. To adjust for multiple pairwise comparisons, the Bonferroni correction was applied to obtain the reported *p*-values. To analyze vesicle distributions containing APPL in the migratory EP cells (**Figure 5C**), confocal images were collected from regions corresponding to trailing and leading cells within each embryo. Maximum intensity projections containing three optical sections (spanning a total of 0.9 μm) were converted to images in Fiji that contained only the overlap in nAPPL and cAPPL signals. The images were then converted to binary mode, and subjected to watershed segmentation. ROIs encompassing individual EP cells were then selected to quantify large perinuclear vesicles containing APPL, using the “analyze particle” command. To normalize the accepted particles across images, particle size was set as 1/100 of the average area for all EP cells in that image. Circularity was set from 0.05 to 1, and quantification was performed for each EP cell both as the number of vesicles per cell and the percent area of large APPL-containing vesicles based on total cell area. Both methods of analysis produced similar results; accordingly, the number of vesicles per EP cell was used for subsequent reporting purposes. Student's two-tailed *t*-tests were used to compare the average number of vesicles in trailing vs. leading cells at each developmental stage. At least three embryos were included at each stage, with a total of 10–20 EP cells averaged in each group.

To analyze fluorescent intensities in the EP cells by quantitative immunofluorescence confocal microscopy (**Figure 8B**), relative intensity values were calculated independently for APPL and Fas II expression in each experimental group (*n* = 10 per group), and statistical differences between groups were calculated using unpaired Student's *t*-tests. One-way ANOVA was used to determine if means differed among the four groups, followed by Student's two-tailed *t*-tests for pairwise comparisons. The Bonferroni correction was applied to obtain reported *p*-values. For our analysis of EP cell migration and outgrowth (**Figure 8D**), means were calculated for each experimental group, normalized to matched control embryos in each preparation (*n* = 10 per condition). Statistical differences between groups were calculated using one-way ANOVA, followed by unpaired Student's *t*-tests with the Bonferroni correction to obtain *p*-values. For our analysis of vesicle movements in cultured *Drosophila* neurons (**Figure 11**), at least 4 events per cell were analyzed per genotype (*n* > 15 cells per genotype) in four independent experiments.

## Results

### Secretase-dependent cleavage of APPL resembles APP processing

Previous work has shown that insect APPL shares the key structural features of mammalian APP_695_ (Figure 1A), while experiments using *Drosophila* have identified orthologs for the three secretase classes associated with APP processing (described below). Notably, secretase-dependent processing of APPL produces the same types of cleavage fragments as APP_695_ (Figure 1A), including soluble ectodomain fragments (sAPPLs), membrane-bound CTFs, intracellular AICD fragments, and a neurotoxic Aβ-like peptide (Luo et al., 1995; Fossgreen et al., 1998; Greeve et al., 2004; Carmine-Simmen et al., 2009; Poeck et al., 2012). To investigate the expression and processing of endogenous APPL in the insect nervous system, we used a panel of antibodies against different domains of *Manduca* APPL (Swanson et al., 2005; Ramaker et al., 2013). In western blots of proteins extracted from developing embryos, larval CNS, and *Manduca* GV1 cells (which endogenously express APPL), antibodies against both C-terminal APPL (Figure 1B) and N-terminal APPL (Figure 1C) recognized the mature holoprotein (135 kDa; black arrows) and a smaller band (~115 kDa; open arrows) that represents an immature, partially glycosylated form. Anti-cAPPL antibodies also recognized a larger band at ~165 kDa in embryonic lysates (open arrowhead), which might represent an additional post-translational modification of APPL that is developmentally regulated (Swanson et al., 2005). In contrast, only anti-nAPPL antibodies labeled a 120 kDa band (Figure 1C, black arrowhead) that was also detected in the medium of cultured GV1 cells (last lane in blot). As previously reported (Swanson et al., 2005), this band represents secreted sAPPL fragments that are cleaved from full-length APPL.

To validate the identities of these bands, we performed cross-immunoprecipitation experiments with *Manduca* embryonic lysates, whereby proteins immunoprecipitated with either anti-cAPPL or anti-nAPPL were immunoblotted with the complementary antibody. As shown in Figures 1D,E, both antibodies specifically recognized the mature and immature forms of the holoprotein (black and open arrows, respectively), whereas the cleaved sAPPL ectodomain fragments (lacking cytoplasmic domains) were not cross-immunoprecipitated. Similar results were obtained when we used other antibodies recognizing different domains of APPL (as indicated in Figure 1A), including anti-Ex-APPL and anti-*Drosophila* APPL 952 (against N-terminal epitopes). As shown in Figure 1F, anti-cAPPL also labeled a pair of candidate CTF fragments (11–15 kDa; black arrows) and a smaller fragment (~8 kDa; open arrow) that represents a candidate AICD fragment. Several anti-APP antibodies targeting C-terminal epitopes that are conserved in APPL (illustrated in Figure 1) also recognized one or both of the CTF bands (not shown), providing additional evidence that these fragments are authentic cleavage products of APPL. Knocking down APPL expression in GV1 cells with morpholino antisense oligonucleotides eliminated labeling of all of these bands (Ramaker et al., 2013, 2016), indicating that they are authentic cleavage fragments of the holoprotein.

These results indicate that *Manduca* APPL is subject to a similar pattern of secretase processing that has been documented for human APP_695_. To explore the identities of the CTF-like fragments detected in *Manduca* lysates, we treated cultured embryos with inhibitors known to block the activity of both insect and mammalian secretases, and then examined the relative abundance of the two bands in western blots labeled with anti-cAPPL (Figure 1G). Using a relatively short exposure time that did not detect the CTFs in control embryos (lane 1), we found that treatment with a γ-secretase inhibitor (DAPT) resulted in a strong elevation in both CTFs (Figure 1G, lane 2), suggesting that endogenously expressed APPL is normally subject to rapid processing (similar to APP; Gralle and Ferreira, 2007; Hare, 2001). Treating embryos with a combination of inhibitors against α- and γ-secretases (Figure 1G, lane 3) resulted in a significant reduction in relative abundance of the larger fragment (the candidate α-CTF of APPL), accompanied by a corresponding increase in the smaller fragment (the candidate β-CTF). Conversely, treatment with a combination of β- and γ-secretase inhibitors selectively reduced the levels of the smaller fragment (Figure 1G, lanes 4). The lower immunoblot in Figure 1G was labeled with anti-actin (Act; ~42 kDa) as a loading control. Quantification of these results are shown in Figure 1H (^*^p = 0.041; ^**^p = 0.0002).

Genetic manipulations of APPL processing in *Drosophila* produced complementary results (Figures 1I,J). Using the eye-specific promoter construct GMR-GAL4, we found that inducing the expression of an additional copy of full-length APPL (via UAS-Appl) resulted in detectable levels of both α-CTFs and β-CTFs (Figure 1I, lane 1). As expected, expressing additional *Drosophila* Presenilin (via UAS-dPsn) reduced both α- and β-CTF levels in these flies (lane 2), verifying that CTFs derived from APPL are subject to secondary processing by γ-secretase activity. In contrast, expressing additional *Drosophila* BACE plus dPsn (via UAS-dBACE plus UAS-dPsn) preferentially reduced α-CTF levels (lane 3), whereas overexpressing the α-secretase Kuzbanian (via UAS-Kuz) caused a marked increase in α-CTF levels, accompanied by the loss of β-CTF (lane 4). In a second experiment, we again showed that expressing additional full-length APPL (via GMR-GAL4) resulted in a marked increase in both α-CTF and β-CTF levels (Figure 1J, lane 1), whereas only the α-CTF band was faintly detectable at this exposure in control flies (carrying GMR-GAL4; lane 2). In contrast, eliminating one copy of Kuzbanian (*kuz/*+) moderately reduced α-CTF levels in these flies (lane 3, upper band), although paradoxically, β-CTF levels were also reduced (lower band). This result may reflect the fact that blocking APPL cleavage by α-secretases also prolongs the retention of the holoprotein in the plasma membrane (described below), which may interfere with its subsequent trafficking into compartments required for β-secretase processing (Nalivaeva and Turner, 2013). In contrast, expressing an additional copy of dBACE caused a marked increase in β-CTF levels, as expected (lane 4). The lower immunoblots in Figures 1I,J was labeled with anti-tubulin (Tub; ~55 kDa) as a loading control.

### APPL trafficking and processing corresponds to the motile behavior of cultured neurons

**Figure 2:**
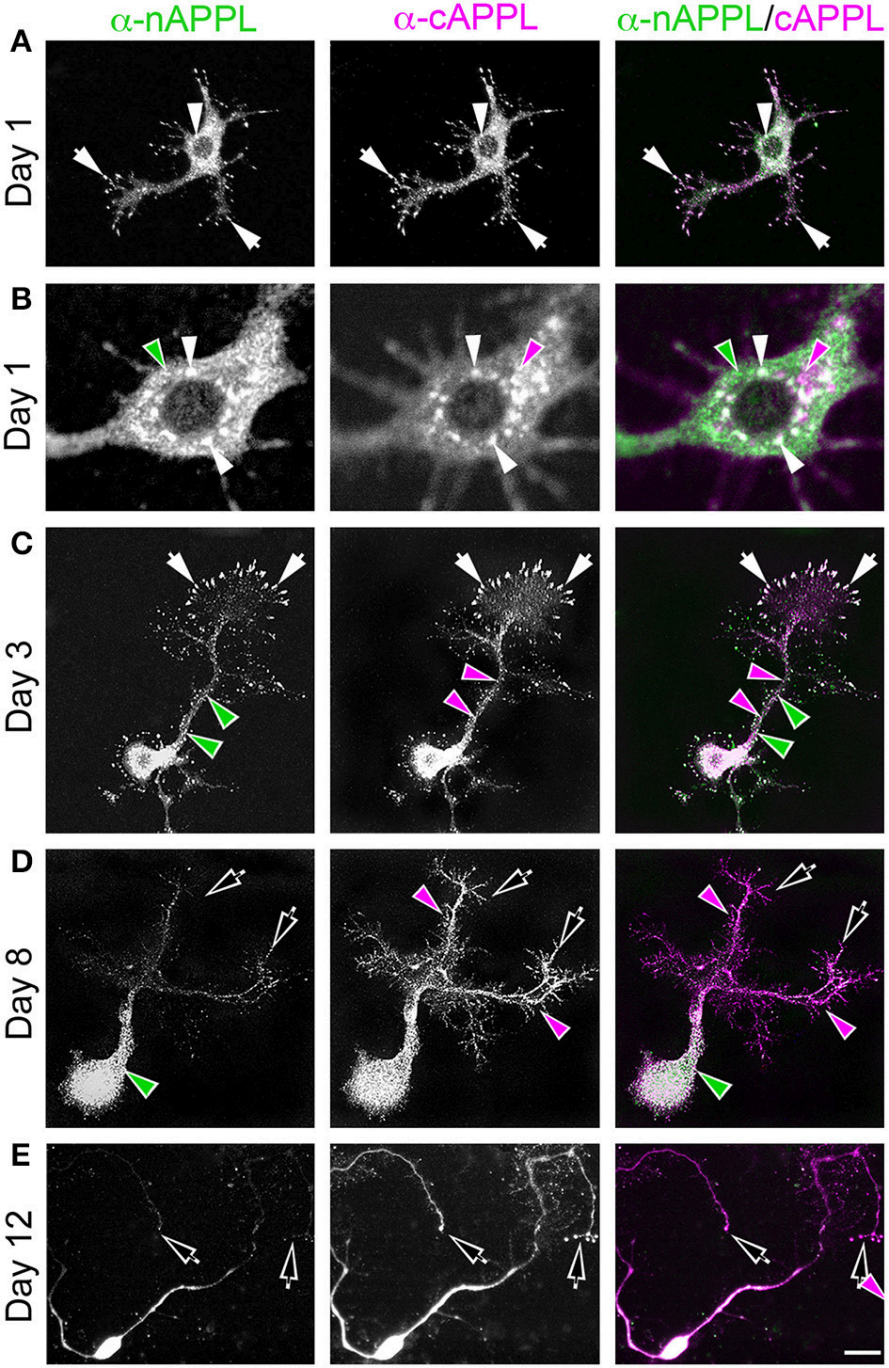
**APPL trafficking and processing in cultured *Manduca* neurons corresponds to their stage of outgrowth**. Neurons harvested from the CNS of fifth instar larvae were grown as dispersed cultures on glass coverslips for 1–14 days *in vitro*, then fixed and immunolabeled with a combination of anti-nAPPL (green) and anti-cAPPL (magenta) antibodies. Anti-nAPPL and anti-cAPPL are shown individually as gray scale images but are shown in green and magenta (respectively) in the merged images, whereby co-immunolabeling appears white (right hand column). **(A)** By Day 1, neurons had begun to extend numerous processes with exploratory growth cones (arrows); full-length APPL (white immunolabeling) accumulated in the leading filopodia, while C-terminal fragments (magenta arrowheads) were relatively more abundant throughout the growing neurites and N-terminal fragments (green arrowheads) were enriched in the cell bodies. APPL holoprotein also accumulated in a population of large perinuclear cytoplasmic vesicles (white arrowhead). **(B)** Higher magnification view of the neuron shown in **(A)**; numerous small vesicles containing only N-terminal fragments (green arrowheads) or C-terminal fragments (magenta arrowhead) were interspersed among the larger vesicles containing the holoprotein (white arrowheads). **(C)** By day 3, many neurons had extended primary neurites with enlarged growth cones; as is apparent in the merged image, APPL holoprotein (white immunolabeling) continued to be enriched at the leading edges of the growth cones and in some filopodia (arrows), while numerous smaller vesicles containing either N-terminal or C-terminal fragments were distributed throughout the neuronal somata and processes. **(D)** By day 8, most neurons were no longer undergoing active outgrowth. Full-length APPL was still abundant within vesicles in their somata (white immunolabeling in the merged image) but was no longer concentrated in the distal tips of their processes (open arrows). By comparison, vesicles containing C-terminal fragments (magenta arrowheads) were diffusely distributed throughout the neurons and their processes, while N-terminal fragments (green arrowheads) remained relatively more abundant in the somata. **(E)** By 12 days, most neurons were either undergoing retraction (open arrows) or initiating degeneration (not shown). APPL holoprotein was almost completely absent from their distal processes, as were vesicles containing N-terminal fragments, whereas vesicles containing C-terminal fragments (magenta) could still be detected throughout the neurons. These results suggest that full-length APPL is selectively transported to the distal tips of developing neurons during periods of active outgrowth. Scale bar = 2 μm in **(B)**; 10 μm in all other panels.

To explore how APP family proteins are developmentally regulated, we first examined the endogenous distributions of APPL in isolated *Manduca* neurons grown in culture. In newly plated neurons that had commenced their initial outgrowth (day 1 *in vitro*; DIV), we could readily detect membrane-associated APPL within the most distal regions of their growing processes (Figure 2A; arrows). Note that anti-nAPPL and anti-cAPPL immunolabeling is presented as gray scale images for the individual channels but shown in green and magenta (respectively) in the merged images, whereby co-immunolabeling appears white (right hand column). However, we also found that a substantial portion of full-length APPL localized to a distinct population of large cytoplasmic vesicles (Figures 2A,B, white arrowheads), which were intermingled with numerous smaller vesicles containing either N-terminal (Figure 2B, green arrowheads) or C-terminal fragments (Figure 2B, magenta arrowheads). This pattern persisted throughout subsequent periods of neurite outgrowth (3–5 DIV): some full-length APPL was concentrated at the distal tips of exploratory growth cones (Figure 2C, arrows), while more of the holoprotein and its fragments localized to different vesicle populations. In general, N-terminal fragments were relatively more abundant in the neuronal somata while C-terminal fragments were enriched in their growing neurites, although we could detect vesicles containing both fragments throughout the neurons.

By comparison, neurons that were no longer actively growing (at 8–10 DIV) exhibited a markedly different pattern of APPL immunoreactivity. Although, full-length APPL was still readily apparent in large cytoplasmic vesicles within their cell bodies, the holoprotein was greatly reduced in their distal processes (Figure 2D; open arrows), while smaller vesicles containing C-terminal fragments remained abundant throughout their neurites. This distinction was even more apparent in neurons undergoing retraction (12–14 DIV), in which the holoprotein was largely absent from their most distal regions (Figure 2E; open arrowheads), leaving only a diffuse distribution of cAPPL immunolabeling in the neuronal processes (shown in magenta). These results indicate that in neurons undergoing active outgrowth, full-length APPL is rapidly transported into their most motile regions but then is rapidly cleaved, whereas in non-motile or retracting neurons, the holoprotein accumulates predominantly in large cytoplasmic vesicles.

### APPL trafficking and processing corresponds to the motile behavior of migratory neurons *In vivo*

**Figure 3:**
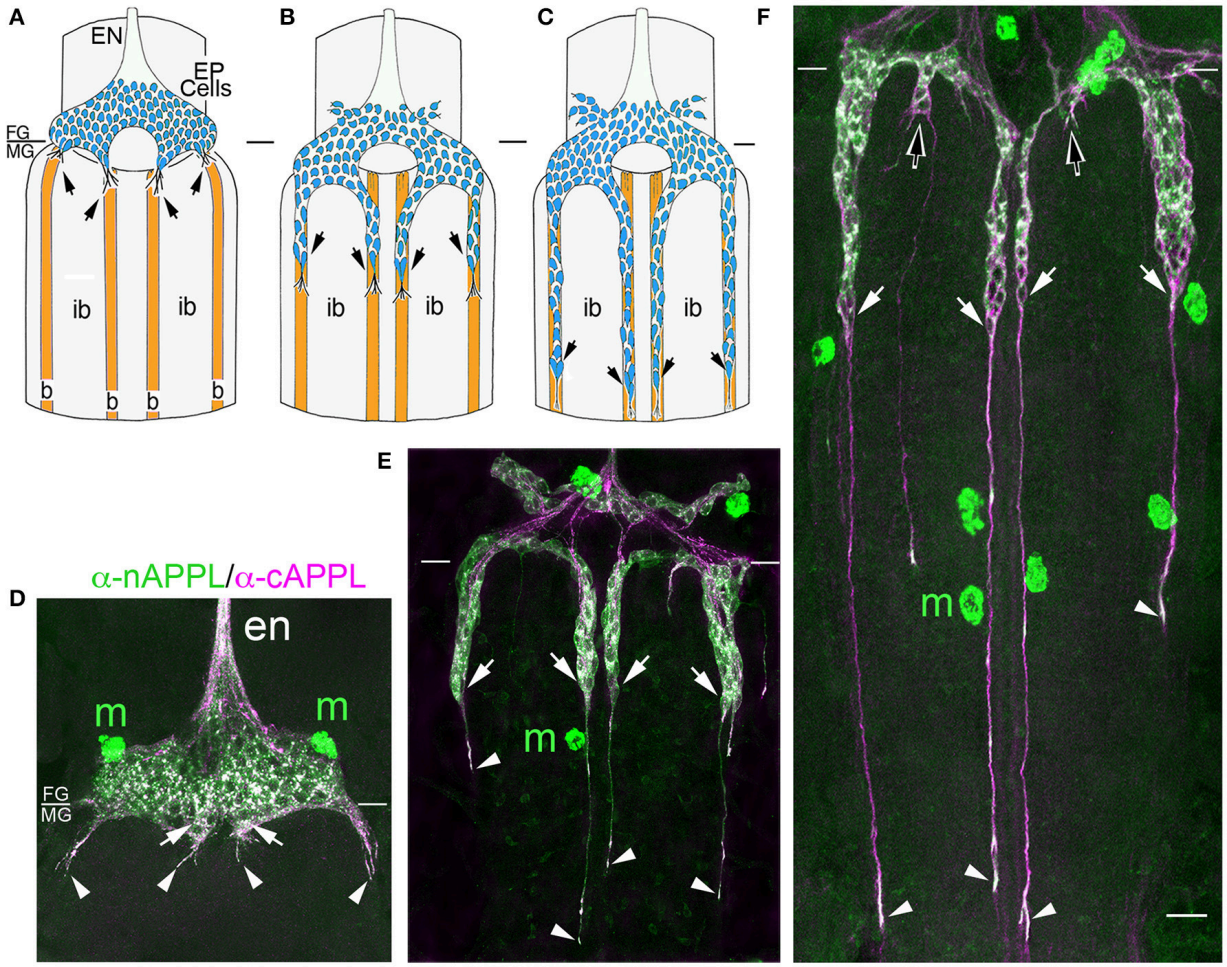
**APPL expression is developmentally regulated by the migratory EP cells within the enteric nervous system (ENS) of *Manduca*. (A–C)** Schematic diagrams of EP cell migration, illustrating the progression of the motile neurons (blue) along the midgut muscle bands (orange). Black arrows indicate the positions of the leading neurons on each band pathway. **(A)** Embryo at 55 HPF; at this stage, the EP cells form a packet of pre-migratory neurons that have spread bilaterally around the circumference of the foregut, adjacent to the foregut-midgut boundary (FG/MG). Small groups of these neurons extend their leading processes onto each of the eight coalescing muscle bands (“b”) on the midgut (only the four dorsal bands are shown). **(B)** By 60 HPF, small groups of EP cells have begun to migrate and extend leading processes along the muscle bands while avoiding the adjacent interband regions (“ib”). **(C)** By 65 HPF, the EP cells have transitioned from active migration to an extended period of outgrowth, during which they elongate fasciculated axons along each band pathway (beyond the field of view in **C**). Only once axon outgrowth is complete (80 HPF) will the neurons extend terminal synaptic processes onto the interband regions (not shown). **(D–F)** Staged *Manduca* embryos fileted to expose the developing ENS and immunolabeled with a combination of anti-nAPPL (green) and anti-cAPPL (magenta) antibodies. **(A)** Embryo at 55 HPF (compare with **A**). APPL is robustly expressed by the EP cells but not the adjacent muscle cells of the foregut and midgut. Full-length APPL (white immunolabeling) accumulates in the leading processes of neurons that have contacted adjacent muscle bands (arrowheads), and also in a population of large cytoplasmic vesicles within the pre-migratory neurons. By comparison, C-terminal fragments (magenta) are relatively more abundant within fasciculated axons in the anterior esophageal nerve of the foregut (“en”), while N-terminal fragments (green) are diffusely localized throughout the somata of the pre-migratory EP cells. N-terminal fragments are also highly concentrated in peripheral macrophage hemocytes (“m”) that surveil the developing nervous system and sequester cleaved APPL ectodomains (unpublished observations). **(D)** Embryo at 60 HPF (compare with **B**). EP cells that have migrated onto the muscle bands continue to exhibit robust levels of APPL expression, with full-length APPL being concentrated in their leading processes and growth cones (arrowheads). At this stage, the leading neurons on each pathway contain substantially fewer large cytoplasmic vesicles enriched with full-length APPL (white immunolabeling) than trailing/stationary neurons. **(E)** Embryo at 65 HPF (compare with **C**); at this stage, the EP cells transition from migration to axon outgrowth. Full-length APPL (white immunolabeling) continues to be concentrated in their leading growth cones (arrowheads). C-terminal APPL fragments (magenta) are noticeably more abundant in their fasciculated axons, while N-terminal fragments (green) are more apparent in their somata (as well as in the peripheral macrophages). Open arrows indicate small subsets of EP cells that occupy foregut nerves between adjacent band pathways and that extend short processes onto the interband musculature. Scale bar = 30 μm.

Previously, we showed that APPL expression is strongly upregulated by migratory neurons that populate the Enteric Nervous System (ENS; Swanson et al., 2005). During the formation of the ENS (Figure 3), a population of ~300 neurons (EP cells) undergoes a stereotyped sequence of migration to form a branching nerve plexus called the Enteric Plexus, which spans the foregut-midgut boundary (FG/MG). After emerging from a neurogenic placode (Copenhaver and Taghert, 1990), the EP cells initially spread bilaterally around the circumference of the foregut (from 40 to 55 HPF; Figure 3A), during which subsets of neurons align with one of eight equivalent muscle bands (“b”) that coalesce on the midgut surface. The neurons then abruptly commence a phase of active migration onto these band pathways (from 55 to 65 HPF; Figure 3B), traveling in a chain-like manner along each band while avoiding adjacent interband regions (“ib”). Subsequently, the neurons transition from migration to axon elongation (at ~65 HPF, Figure 3C), during which they extend fasciculated bundles of axons posteriorly along the muscle bands. Only after axon outgrowth is complete do the EP cells elaborate terminal branches onto the adjacent interband musculature (from 80 to 100 HPF), providing a diffuse innervation to the midgut (Copenhaver and Taghert, 1989a,b). Accordingly, we used this preparation to examine the developmental regulation of APPL in motile neurons within the developing nervous system.

**Figure 4:**
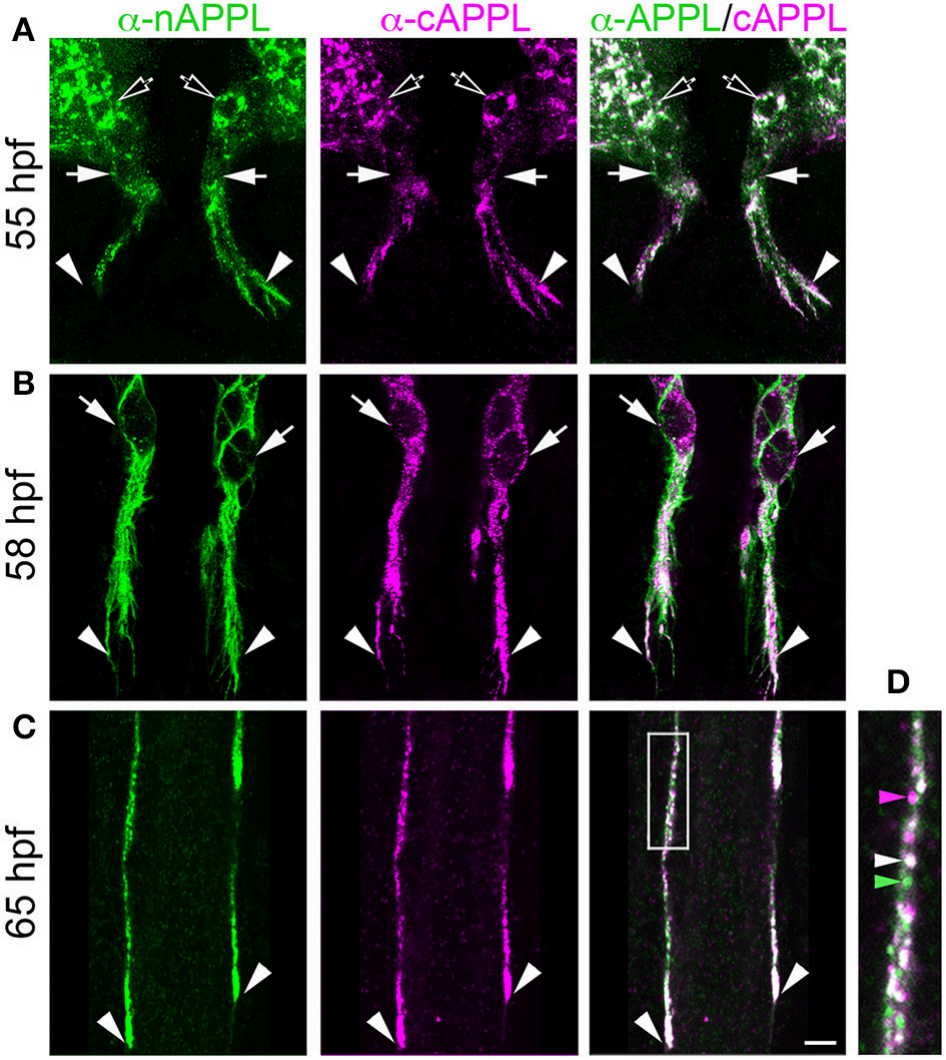
**Full-length APPL traffics into the leading growth cones and elongating axons of the motile EP cells. (A–C)** show the developing ENS of fileted embryos at progressive stages of development, double-immunolabeled with antibodies against nAPPL (green) and cAPPL (magenta) epitopes. Arrowheads indicate leading growth cones/processes; arrows indicate leading EP cell bodies. **(A)** 55 HPF: in EP cells that have begun to migrate, full-length APPL (white immunolabeling) is concentrated in their leading processes rather than their cell bodies. In contrast, trailing neurons that have not begun to migrate (open arrowheads) exhibit numerous large vesicles containing the holoprotein. **(B)** 58 HPF: in EP cells undergoing active migration (arrows), full-length APPL continues to be concentrated in their leading processes (arrowheads), with relatively few large vesicles in their somata. **(C)** 65 HPF: once the EP cells have transitioned to axon outgrowth, full-length APPL continues to accumulate in their growth cones that extend posteriorly along the midgut (arrowheads). **(D)** Magnified view of boxed region in **(C)**, revealing intermingled populations of smaller vesicles containing only nAPPL fragments (green arrowhead), cAPPL fragments (magenta arrowhead), or both epitopes (white arrowhead). Scale bar = 5 μm in **(A–C)**, 1.5 μm in **(D)**.

For our initial analysis, we used a combination of antibodies specific to the N- and C-terminal domains of APPL to distinguish the holoprotein from its fragments in immunolabeled embryos. Intriguingly, we observed a similar sequence of endogenous APPL trafficking and processing in the migratory EP cells as seen in cultured neurons, corresponding to their motile behavior. In pre-migratory neurons (55 HPF), we could readily detect full-length APPL in their leading processes contacting their future band pathways (Figure 3D, arrowheads), and subsequently in the growth cones of their axons extending posteriorly along the band pathways (Figures 3E,F, arrowheads). Figure 4 shows magnified views of how full-length APPL continues to be concentrated within the leading processes of the EP cells throughout their migration and subsequent axon outgrowth. As in our primary neuronal cultures, we also consistently detected abundant populations of smaller vesicles labeled with anti-nAPPL, anti-cAPPL, or both domains (highlighted in Figure 4D). Once again, vesicles containing only N-terminal epitopes (green) tended to be smaller in diameter and were more abundant in the cell bodies, while larger vesicles containing only C-terminal epitopes (magenta) were more abundant in their elongating axons (Figure 3F). Also apparent at lower magnification were hemocyte macrophages that labeled only with anti-nAPPL antibodies (Figures 3D–F, “m”). In unpublished studies, we have used proteomics methods and qRT-PCR assays to show that these macrophages do not themselves express full-length APPL; rather, they sequester cleaved sAPPL ectodomains both in primary culture and in cultured embryos, indicating that they scavenge fragments released by the developing neurons. Of note is that we obtained similar results using different fixation and permeabilization conditions (including 2–8% PFA, Bouin's and Zamboni's fixatives), and with a variety of antibodies against different N- and C-terminal epitopes within APPL.

**Figure 5:**
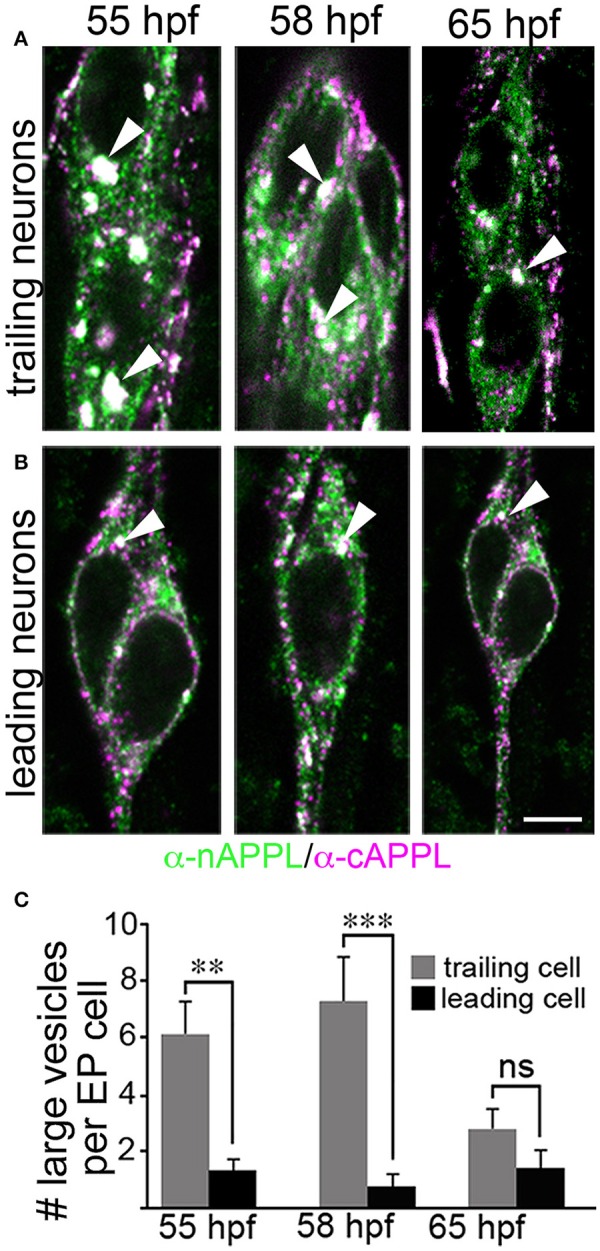
**The cytoplasmic distribution of full-length APPL changes with the motile behavior of developing neurons**. Magnified views of EP cells at progressive stages of embryogenesis, double- immunolabeled with antibodies against nAPPL (green) and cAPPL (magenta) epitopes. **(A)** Trailing EP cells that had not yet begun to migrate (55–58 HPF) or that had transitioned from migration to axon outgrowth (65 HPF). **(B)** Leading EP cells that were undergoing active locomotion (55–58 HPF) or that had transitioned from migration to axon outgrowth (65 HPF). Arrowheads indicate examples of the large vesicles labeled with both nAPPL and cAPPL antibodies (white immunolabeling); this vesicle population was markedly more abundant in the non-migratory neurons. **(C)** Quantification of the number of large vesicles (>100 nm) that apparently contain full-length APPL. At 55 and 58 HPF, the number of large vesicles was significantly reduced in leading EP cells, compared to trailing EP cells (^**^p = 0.002 and ^***^p = 0.001, respectively; ns = not significant). At 65 HPF, there was no significant difference in the number of large perinuclear vesicles in trailing vs. leading EP cells (*p* = 0.106). Statistical comparisons were performed using pairwise Student's two-tailed *t*-tests; *N* ≥ 10 per group; histograms show means ± SD. Scale bar in **(A,B)** = 5 μm.

However, as with our neuronal cultures, we also observed that much of the holoprotein did not reside at the plasma membrane, accumulating instead within large cytoplasmic vesicles that co-immunolabeled with antibodies against both N- and C-terminal APPL (visible as white vesicles in Figures 3, 4). This vesicle class was particularly abundant in EP cells that had not yet begun to migrate (Figure 4A, open arrows) but became noticeably reduced in neurons undergoing active locomotion (Figures 4A,B, arrows), coincident with the accumulation of the holoprotein in their leading processes (Figure 4B, arrowheads). This developmentally regulated redistribution of APPL was more apparent at higher magnification (Figure 5). When we compared the number of large vesicles (>100 nm) in migratory EP cells vs. trailing/stationary neurons at progressive stages of development, we found that the leading neurons on each pathway (undergoing active locomotion) had significantly fewer of these large vesicles (Figure 5B, 55–58 HPF) than in trailing neurons that had not yet begun to migrate (Figure 5A, 55–58 HPF). Even after the neurons had transitioned from migration to axon outgrowth (65 HPF), the relative abundance of these vesicles remained low, while full-length APPL continued to accumulate in the growth cones of their elongating axons (Figure 4C, arrowheads). Quantification of large vesicles in leading vs. trailing neurons at each developmental stage is shown in Figure 5C (^**^p = 0.002; ^***^p = 0.001; ns = not significant).

**Figure 6:**
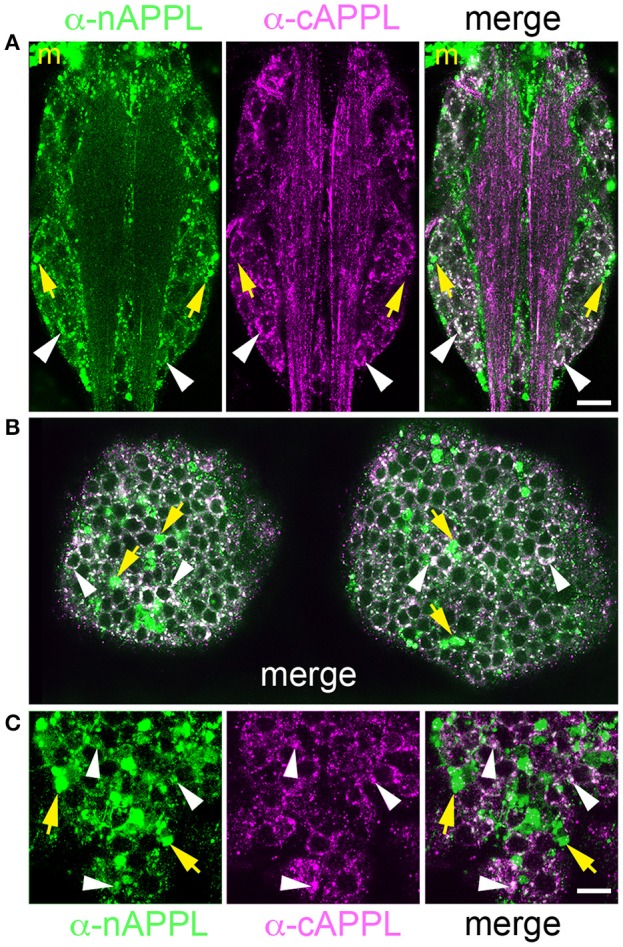
**Different cleavage fragments of APPL are concentrated within distinct domains of neurons in the developing *Manduca* CNS. (A)** Abdominal ganglion of an embryo (at 60 HPF) that was immunolabeled with a combination of anti-APPL antibodies. As in the EP cells, large cytoplasmic vesicles containing APPL holoprotein were abundant in most neurons (white arrowheads). In addition, anti-nAPPL labeling (green) was more abundant in the neuronal somata (located in the cortical regions of the ganglia), whereas anti-cAPPL (magenta) was more abundant in both the somata and their processes within the central neuropil regions, including prominent fascicles of longitudinal axons. Anti-nAPPL antibodies also immunolabeled peripheral macrophages (“m”) that do not themselves express APPL but rather scavenge cleaved ectodomain fragments released by neurons (unpublished observations). Similarly, an additional population of cells within the ganglia immunolabeled only with anti-nAPPL but not anti-cAPPL (yellow arrowheads). **(B)** Embryonic brain from the same developmental stage; the large cytoplasmic vesicle population containing APPL holoprotein was apparent in most neuronal somata (white arrowheads), interspersed with smaller vesicles containing either nAPPL or cAPPL fragments. **(C)** Magnified view of the brain shows neurons with vesicles containing the holoprotein (white arrowheads), intermingled with smaller cells that were labeled only with anti-nAPPL (yellow arrows). Scale bar = 30 μm in **(A,B)**; 7 μm in **(C)**.

We detected a similar pattern of APPL fragments within the developing CNS (Figure 6). In both segmental ganglia (Figure 6A) and brain (Figures 6B,C), vesicles containing only N-terminal APPL fragments (green) were considerably more abundant in the neuronal somata (occupying cortical regions of the CNS), while vesicles containing only C-terminal fragments (magenta) were enriched within more central neuropil regions and axon fascicles. Most neurons also contained the larger class of cytoplasmic vesicles that labeled with both N- and C-terminal antibodies, indicating the presence of the holoprotein (Figure 6, white arrowheads). As in the developing ENS, we routinely observed peripheral macrophages associated with the surface of the CNS that labeled only with anti-nAPPL antibodies (Figures 6A–C, “m”), due to their sequestration of cleaved sAPPL fragments released from the nervous system (unpublished observations). In addition, we detected a distinct population of smaller cells in the CNS (yellow arrowheads) that were also labeled with anti-nAPPL but not anti-cAPPL. As discussed below, these cells might represent an astrocyte-like population that also scavenges sAPPL fragments or they may comprise a distinct neuronal subtype that preferentially accumulates nAPPL cleavage products in their somata.

### APPL localizes to an amphisome-like compartment in a developmentally regulated manner

**Figure 7:**
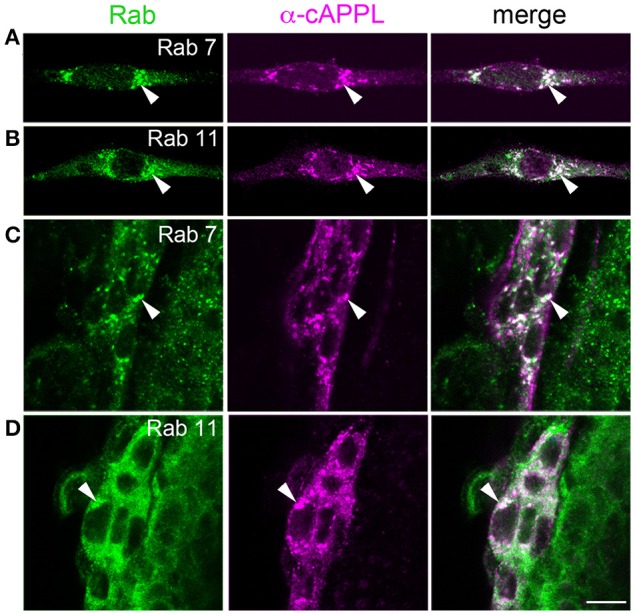
**APPL holoprotein is concentrated in an amphisome-like compartment in GV1 cells and EP cells**. **(A,B)** Examples of *Manduca* GV1 cells that were fixed and immunolabeled with a combination of antibodies against different Rab proteins (shown in green) and APPL (only anti-cAPPL is shown; magenta). Both anti-Rab7 **(A)** and anti-Rab11 **(B)** co-label a population of large cytoplasmic vesicles containing APPL holoprotein (arrowheads), similar to the vesicles found in developing neurons. **(C,D)** Examples of migrating EP cells in fixed embryos (60 HPF) that were immunolabeled with the same combinations of antibodies. Both anti-Rab7 **(C)** and anti-Rab11 **(D)** co-label the large cytoplasmic vesicles containing APPL holoprotein (arrowheads). Scale bar = 7 μm.

To explore the identity of the large vesicles that accumulate APPL, we initially used *Manduca* GV1 cells, which (as noted above) endogenously express APPL. For this analysis, we used a panel of antibodies against proteins associated with different intracellular compartments that had been previously shown to label the targeted proteins in *Drosophila* or that were directed against evolutionarily conserved motifs. Surprisingly, although antibodies against Rab4, Rab5, Rab9, and Rab10 clearly labeled different vesicle populations in GV1 cells (Supplementary Figure S1), none of these compartment markers colocalized with the large vesicles containing APPL holoprotein. Likewise, we detected little or no colocalization of APPL with markers for Golgi (anti-*Drosophila* Lava lamp), mitochondria (Mitotracker Green), lysosomes (anti-Lamp1 and Lysotracker Red), or proteins associated with multivesicular bodies (VPS4) and exosomes (Evi) (Supplementary Figure S1; and data not shown). In contrast, multiple antibodies against both Rab7 and Rab11 strongly co-labeled APPL-positive vesicles in GV1 cells (Figures 7A,B), and this finding was recapitulated in the migratory EP cells (Figures 7C,D). Although, Rab7 is typically associated with late endosomes while Rab11 is associated with recycling endosomes (Stenmark, 2009; Hutagalung and Novick, 2011), recent studies have shown that Rab7 and Rab11 converge during the formation of amphisomes, a large intermediate organelle that gives rise to autophagosomes but also participates in a variety of developmental and signaling functions (Patel et al., 2013; Sanchez-Wandelmer and Reggiori, 2013; Szatmari et al., 2014; Bader et al., 2015). These observations suggest that APPL may be alternatively trafficked either into regions of active growth (where it can modulate motile responses) or into an amphisome-like compartment (for subsequent redistribution and/or processing), providing a previously unrecognized mechanism for regulating the developmental distribution of APP family proteins in embryonic neurons.

### Secretase inhibitors alter both APPL trafficking and neuronal migratory behavior

In previous work, we demonstrated that APPL plays an important role as a neuronal guidance receptor that interacts with heterotrimeric G protein Goα within the leading processes of the EP cells (Swanson et al., 2005; Ramaker et al., 2013). We also showed that APPL-Goα signaling normally prevents the neurons from growing inappropriately into the interband regions of the midgut: inhibiting APPL expression or Goα activation permitted ectopic migration and outgrowth, whereas hyperactivation of this response caused a collapse/stall response that blocked normal migration (Horgan and Copenhaver, 1998; Ramaker et al., 2013, 2016). Based on these results, we hypothesized that the accumulation of APPL within the leading processes of the EP cells must be precisely regulated to prevent hyperactivation of APPL-Goα signaling, whereby secretase-dependent cleavage of transmembrane APPL would provide a mechanism for terminating APPL-dependent responses (thereby preventing inappropriate collapse-stall behaviors).

A prediction from this model is that preventing the normal cleavage of membrane-associated APPL should increase the relative abundance of APPL in the EP cell membranes and enhance the normal activation of APPL-dependent retraction responses. As already noted, treating *Manduca* embryos with inhibitors targeting α-, β-, and γ-secretases caused predictable changes in the cleavage of the holoprotein (Figure 1G). Accordingly, we used our embryo culture assay to test whether inhibiting different aspects of APPL processing in the EP cells also affected their migratory behavior. For these experiments, we treated cultured embryos just after the onset of EP cell migration (at 57 HPF) with specific secretase inhibitors for 5 h, then fixed and immunolabeled the preparations with anti-nAPPL, anti-cAPPL, and anti-Fas II (as an independent membrane marker). We then quantified both the relative levels of membrane-associated APPL and the extent of EP cell migration and outgrowth.

**Figure 8:**
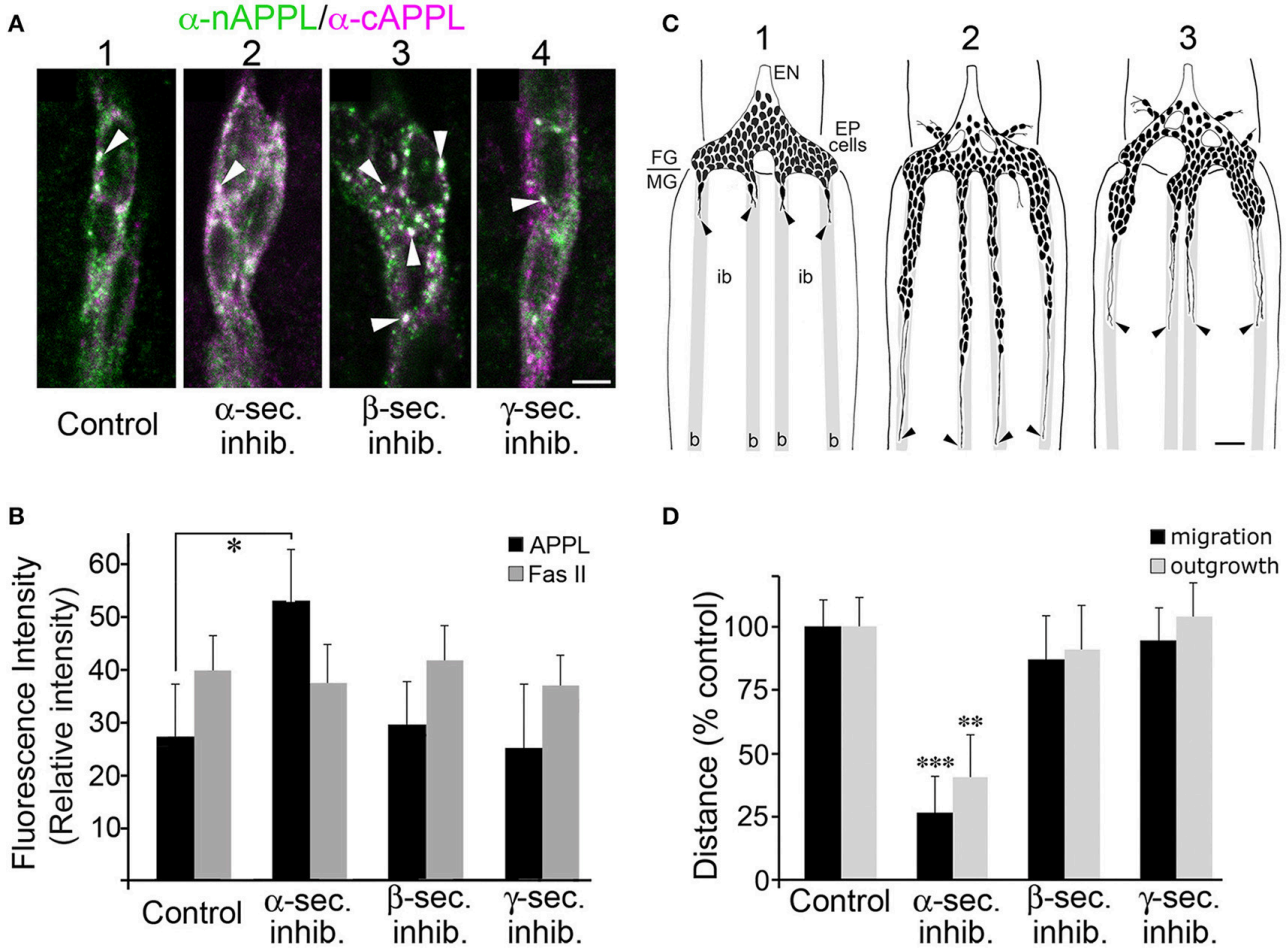
**Blocking α-secretase activity increases membrane-associated APPL levels in the EP cells and inhibits their migration. (A)** Examples of EP cells in cultured embryos that were treated with different secretase inhibitors and then immunolabeled with a combination of anti-nAPPL (green) and anti-cAPPL antibodies (magenta). **(A**_1_**)** EP cells in a cultured control preparation. **(A**_2_**)** EP cells treated with an α-secretase inhibitor showed increased levels of membrane-associated full-length APPL (white). **(A**_3_**)** EP cells treated with a β-secretase inhibitor showed an increased number of cytoplasmic vesicles containing the holoprotein (arrowheads). **(A**_4_**)** EP cells treated with a γ-secretase inhibitor showed an apparent increase in C-terminal fragments (magenta). **(B)** Quantification of the relative amount of membrane-associated APPL (black histograms) in EP cells treated with different secretase inhibitors (normalized to adjacent interband regions in each preparation). Treatment with α-secretase inhibitors caused a noticeable increase in membrane APPL that was significant in a pairwise comparison (^*^p < 0.02), but not quite significant after applying the Bonferroni correction for multiple comparisons (*p* < 0.06). In contrast, none of the other secretase inhibitors affected the relative levels of APP, nor was the intensity of Fas II immunoreactivity altered by any of these treatments (quantified in a separate channel; gray histograms). Statistical comparisons between groups were performed using one-way ANOVA followed by unpaired Student's two-tailed *t*-tests with the Bonferroni correction to obtain reported *p*-values. *N* = 10 per group; histograms show means ± SD. **(C)** Examples of EP cell migration in cultured embryos (redrawn from *camera lucida* images of immunolabeled preparations). **(C**_1_**)** Embryo that was fixed and immunolabeled at experimental onset (57 HPF); at this stage, the pre-migratory EP cells extended short exploratory processes onto the midgut band pathways (“b”) but avoid the adjacent interband regions (“ib”). **(C**_2_**)** Control preparation that was allowed to develop in culture for 18 h; the EP cells had migrated and extended axons posteriorly along the muscle band pathways (black arrowheads). **(C**_3_**)** Preparation that was treated with an α-secretase inhibitor; EP cell migration and axon outgrowth were markedly reduced compared to controls, although there was no apparent increase in ectopic migration or neuronal death. **(D)** Quantification of the extent of EP cell migration (black histograms) and outgrowth (gray histograms) along the midgut band pathways in cultured embryos treated with different secretase inhibitors (distances normalized to controls in each experimental group). Treatment with α-secretase inhibitors caused a significant reduction in both migration (^***^p < 0.001) and outgrowth (^**^p = 0.002), whereas treatment with β- and γ-secretase inhibitors had no apparent effects on these aspects of EP cell development. Migration and outgrowth distances were normalized to mean values obtained from matched control preparations in each experiment. Statistical comparisons between groups were performed using one-way ANOVA followed by unpaired Student's two-tailed *t*-tests with the Bonferroni correction to obtain reported *p*-values. *N* ≥ 16 per group; histograms show means ± SD. Scale bar in **(A)** = 5 μm; in **(C)** = 40 μm.

Consistent with the images shown in Figures 3, 4, we found that EP cells in cultured control preparations showed a moderate level of full-length APPL at the membrane (Figure 8A_1_; immunolabeled white), plus the same spectrum of cytoplasmic vesicles containing the holoprotein (arrowhead), N-terminal fragments (green), or C-terminal fragments (magenta). In contrast, when we treated the EP cells with α-secretase inhibitors, we detected an increase in full-length APPL immunoreactivity at the plasma membrane (Figures 8A_2_, 8B), consistent with other evidence that α-secretases predominantly cleave APP family holoproteins at the cell surface (Sisodia, 1992; Zhang et al., 2012).

In contrast, inhibiting β-secretase activity caused no significant change in membrane associated APPL (Figure 8B) but did markedly increase the number of large cytoplasmic vesicles containing the holoprotein (Figure 8A_3_, arrowheads). Given our evidence that this vesicle class represents Rab7/Rab11-positive amphisomes (Figure 7), these data suggest that a substantial portion of full-length APPL normally traffics into this compartment in a developmentally regulated manner, where it is cleaved by β-secretase. Interestingly, treatment with γ-secretase inhibitors also did not significantly alter the levels of full-length APPL at the plasma membrane (Figure 8B) but did produce a noticeable increase in C-terminal fragments (Figure 8A_4_, magenta). This result indicates that low basal γ-secretase activity normally removes CTFs from the neuronal membranes following α-secretase cleavage, consistent with current models that γ-secretase processing of APP occurs only after initial cleavage by α- or β-secretases (Turner et al., 2003; Zhang et al., 2012). By comparison, Fas II levels were not significantly affected by any of the secretase treatments (Figure 8B, gray histograms).

When we subsequently analyzed the motile behavior of the EP cells in these preparations, we found that treatment with α-secretase inhibitors resulted in a significant inhibition of both migration and outgrowth (Figure 8C), although we observed no obvious changes in neuronal viability. Quantification of these results are shown in Figure 8D (^**^p = 0.002; ^***^p < 0.001; determined using unpaired Student's two-tailed *t*-tests with the Bonferroni correction). In contrast, inhibitors targeting β- and γ-secretases had no detectable effect on migration and outgrowth (Figure 8D), indicating that altered CTF and AICD levels did not perturb EP cell development over this 5-h culture period. These results support the model that increased APPL levels in the leading processes of the EP cells permits exaggerated activation of APPL-Goα signaling, which in turn restricts normal migration.

### Trafficking and processing of fluorescently tagged APPL and APP in cultured *Drosophila* neurons

**Figure 9:**
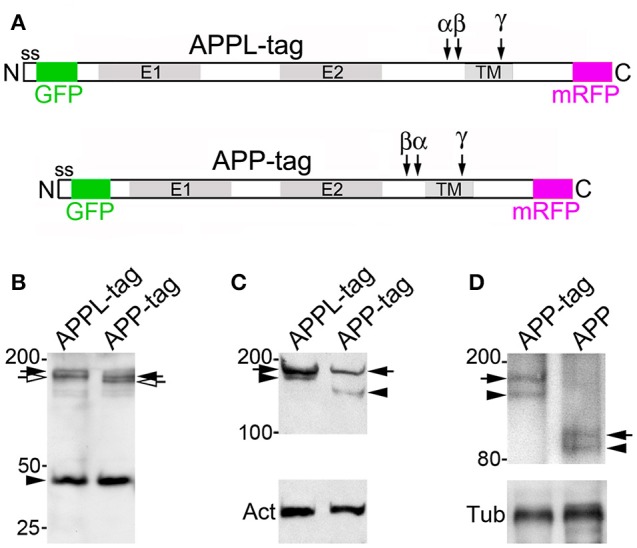
**Expression and processing of fluorescently double-tagged APPL and APP_695_ in *Drosophila*. (A)** Schematic diagram of constructs encoding full-length *Drosophila* APPL and human APP_695_, in frame with N-terminal enhanced Green Fluorescent Protein (GFP; inserted downstream of their signal sequence) and C-terminal monomeric Red Fluorescent Protein (mRFP). E1 and E2 indicate extracellular protein interaction domains; “ss” indicates signal sequences; TM indicates transmembrane domains (compare with Figure 1). Cleavage sites for α, β-, and γ-secretases are indicated by arrows; note that the relative positions of the α and β cleavage sites in APPL are reversed, compared to APP_695_. **(B)** Western blot of head lysates from flies expressing double-tagged APPL (APPL-tag) and APP_695_ (APP-tag) under the control of the eye-specific promoter construct GMR-GAL4. Immunoblot was labeled with anti-DsRed (targeting mRFP). The full length, mature forms (black arrows) and partially glycosylated immature forms (open arrows) of both constructs were expressed at similar levels. Smaller bands at ~42 kDa represent CTF fragments generated by α- and β-secretase cleavage (not distinguished in this gel). **(C)** Western blot of head lysates from the same fly lines immunolabeled with anti-GFP; full-length mature forms (black arrows) and cleaved ectodomain fragments (black arrowheads) of both constructs could be readily detected. The smaller size of cleaved ectodomains from double-tagged APP_695_ reflects the presence of larger intervening sequences between the E1 and E2 domains in *Drosophila* APPL (illustrated in **A**). Lower gel shows anti-actin staining (“Act”) as a loading control. **(D)** Western blot of head lysates from flies expressing either double-tagged APP_695_ (APP-tag) or untagged full-length APP_695_ (APP), labeled with an antibody specific for the N-terminus of human APP. Upper bands (black arrows) indicate full-length, mature holoprotein; lower bands (arrowhead) represent cleaved ectodomain fragments. The larger size of the bands in the APP-tag lane is consistent with the combined molecular weight of APP_695_ plus mRFP and eGFP. Lower gel shows anti-tubulin staining (“Tub”) as a loading control.

To complement our immunohistochemical analysis of APPL in *Manduca*, we also expressed constructs encoding a fluorescently double-tagged version of *Drosophila* APPL in the fly CNS, using the GAL4/UAS system (Brand and Perrimon, 1993). For this experiment, the sequence encoding enhanced Green Fluorescence Protein (eGFP) was inserted immediately after the signal sequence of APPL, while the sequence encoding monomeric Red Fluorescence Protein (mRFP) was inserted in-frame with the C-terminus of the coding domain (Figure 9A). After inducing its expression in the developing eye (using GMR-GAL4), we could readily detect the full-length double-tagged protein (APPL-tag) in western blots of head lysates labeled with anti-DsRed (Figure 9B, lane 1; black arrow); the slightly smaller band (open arrow) may represent a partially glycosylated immature form (Weidemann et al., 1989; Swanson et al., 2005). We could also detect a candidate CTF band at ~42 kDa, consistent with the predicted size of endogenous CTFs (~12–15 kDa) plus C-terminal mRFP, although we could not resolve α-CTFs from β-CTFs in this assay. Likewise, in western blots labeled with anti-GFP (Figure 9C, lane 1), we could detect both full-length (arrow) and cleaved ectodomain fragments (arrowhead) of double-tagged APPL (APPL-tag), recapitulating our analysis of endogenously expressed APPL in *Manduca* (Figures 1B,C). In the lower gel, “Act” indicates actin as a loading control.

Using similar methods, we also expressed a double-tagged construct of human APP_695_ in *Drosophila* neurons (Figure 9A; APP-tag). As with our APPL construct, we could detect both full length and immature forms of double-tagged APP_695_ in western blots of head lysates labeled with anti-mRFP (Figure 9B, lane 2), as well as candidate CTF fragments at ~42 kDa (arrowhead). Likewise, we could detect both the full-length construct and cleaved ectodomain fragments in immunoblots labeled with anti-GFP (Figure 9C, lane 2). The smaller size of the GFP-tagged ectodomain fragments derived from APP_695_ (black arrowhead) reflects the fact that fly APPL contains larger intervening sequences between its E1 and E2 extracellular domains (Figure 1A). “Act” in lower gel indicates actin as a loading control. As an additional control, we compared the expression of our double-tagged APP_695_ construct with an untagged version of APP_695_ in western blots labeled with an antibody specific for N-terminal APP. As shown in Figure 9D, we could detect the full-length forms (arrows) and cleaved ectodomain fragments (arrowheads) of both constructs at their predicted sizes. “Tub” in lower gel indicates tubulin as a loading control. These results demonstrate that our double-tagged constructs of both APPL and APP_695_ undergo similar patterns of secretase cleavage as seen with endogenously expressed APPL (Figure 1).

**Figure 10:**
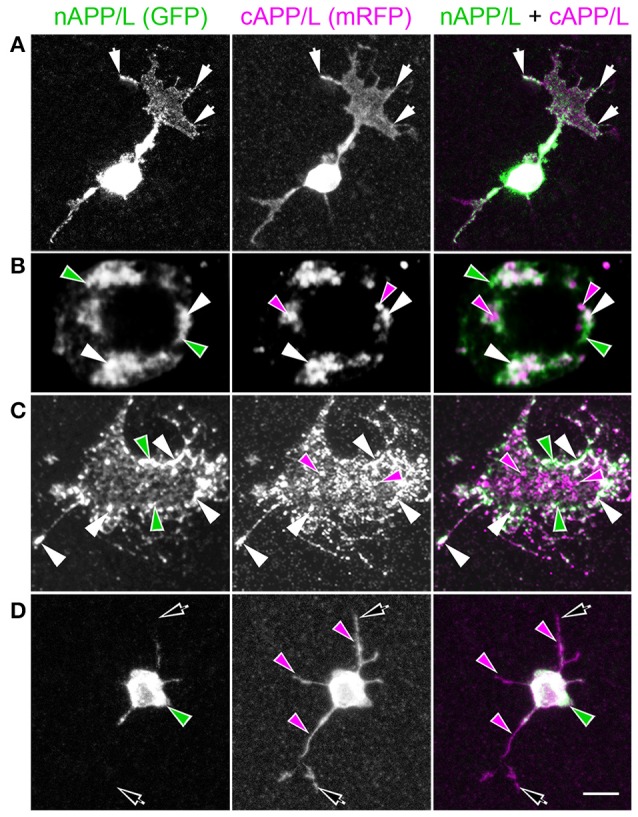
**Trafficking and processing of double-tagged APPL in cultured *Drosophila* neurons corresponds to their stage of outgrowth**. Neurons harvested from the CNS of *Drosophila* white pupae expressing fluorescently double-tagged APPL were grown on coverslips for 1–8 days before fixation and imaging. GFP (nAPPL) and mRFP (cAPPL) are shown individually as gray scale images but are shown in green and magenta (respectively) in the merged images, whereby co-immunolabeling appears white (right hand column). **(A)** In neurons that had recently commenced outgrowth (1 DIV), full-length APPL could be detected in the leading tips of their growth cones (arrows), similar to the distribution of endogenous APPL in cultured *Manduca* neurons (see Figure 2A). In addition, N-terminal fragments (green) were relatively more abundant in their cell bodies, while C-terminal fragments (magenta) were detected throughout the neurons and their processes. **(B)** Higher magnification view of the cell body of a cultured neuron at 2 DIV. Distinct vesicle populations could be detected that contained either nAPPL fragments (green arrowheads) or cAPPL fragments (magenta arrowheads), while an additional population of larger vesicles apparently contained the holoprotein (white arrowheads). **(C)** Higher magnification view of the growth cone of a neuron at 2 DIV. Vesicles containing only nAPPL fragments (green arrowheads) were more concentrated in the peripheral domain, in the vicinity of the holoprotein at the leading margins of the growth cone white arrowheads), while vesicles containing cAPPL fragments (magenta arrowheads) were relatively more abundant within the central domain. **(D)** Older neuron that was undergoing retraction (at 6 DIV); full-length APPL was no longer detectable within its distal processes (open arrows), and N-terminal fragments were largely confined to the cell body (green arrowheads), leaving a diffuse distribution of C-terminal fragments throughout the neurites (magenta arrowheads). These results suggest that the holoprotein is preferentially transported to the distal processes of developing neurons during periods of active outgrowth. Scale bar = 5 μm in **(A,D)**; 1.5 μm in **(B)**, 1 μm in **(C)**.

Accordingly, we expressed these constructs in the CNS and prepared primary cultures of brain neurons (Figure 10). In some preparations, we subsequently fixed and immunolabeled the neurons with anti-GFP and anti-DsRed to enhance the detection of N-terminal cleavage fragments (carrying only the eGFP tag; green) vs. C-terminal fragments (carrying only the mRFP tag; magenta) and the holoprotein containing both tags (visualized as white in merged images). As in Figure 2, nAPPL (GFP) and cAPPL (mRFP) in Figure 10 are presented as gray scale images for the individual channels but shown in green and magenta (respectively) in the merged images, whereby co-localization appears white (right hand column). Notably, we observed that double-tagged APP_695_ and APPL were distributed in a pattern that coincided with stages of outgrowth and retraction (Figure 10), closely matching the patterns that we observed for endogenous APPL in cultured *Manduca* neurons (Figure 2). We could readily visualize the full-length proteins within the leading processes of neurons undergoing active outgrowth (at 1 DIV; Figure 10A, arrows). At higher magnification (Figure 10B), we could also distinguish distinct populations of vesicles that contained either N-terminal fragments (presumably sAPP/sAPPL ectodomains; green arrowheads), C-terminal fragments (including CTFs and AICDs; magenta arrowheads), as well as larger vesicles containing the holoprotein (white arrowheads). Within their growth cone regions (Figure 10C), vesicles containing only N-terminal fragments (green arrowheads) were more concentrated near their leading edges, often in the vicinity of membrane-associated holoprotein (white arrowheads), whereas vesicles containing only C-terminal fragments (magenta arrowheads) were more concentrated within more central domains of the growth cones and proximal neurites. By comparison, in neurons that had ceased to extend processes or were undergoing retraction, little or no full-length protein was detectable in their distal processes (Figure 10D), leaving a diffuse distribution of C-terminal fragments (magenta arrowheads) throughout their neurites while the holoprotein accumulated in their cell bodies, similar to the pattern of APPL fragments seen in retracting *Manduca* neurons (Figure 2E). In some neuronal somata, we also could detect C-terminal fragments within their nuclei (Supplementary Figure S2, arrowhead), consistent with recent evidence that AICD fragments can regulate transcriptional responses in mammalian cells (Cao and Südhof, 2001; Pardossi-Piquard and Checler, 2012) and potentially in the fly brain (Khanna and Fortini, 2015). These results demonstrate that the developmental regulation of our double-tagged constructs closely matches the pattern seen for endogenously expressed APPL.

### Fluorescently tagged APP family proteins exhibit complex patterns of dynamic trafficking in cultured *Drosophila* neurons

**Figure 11:**
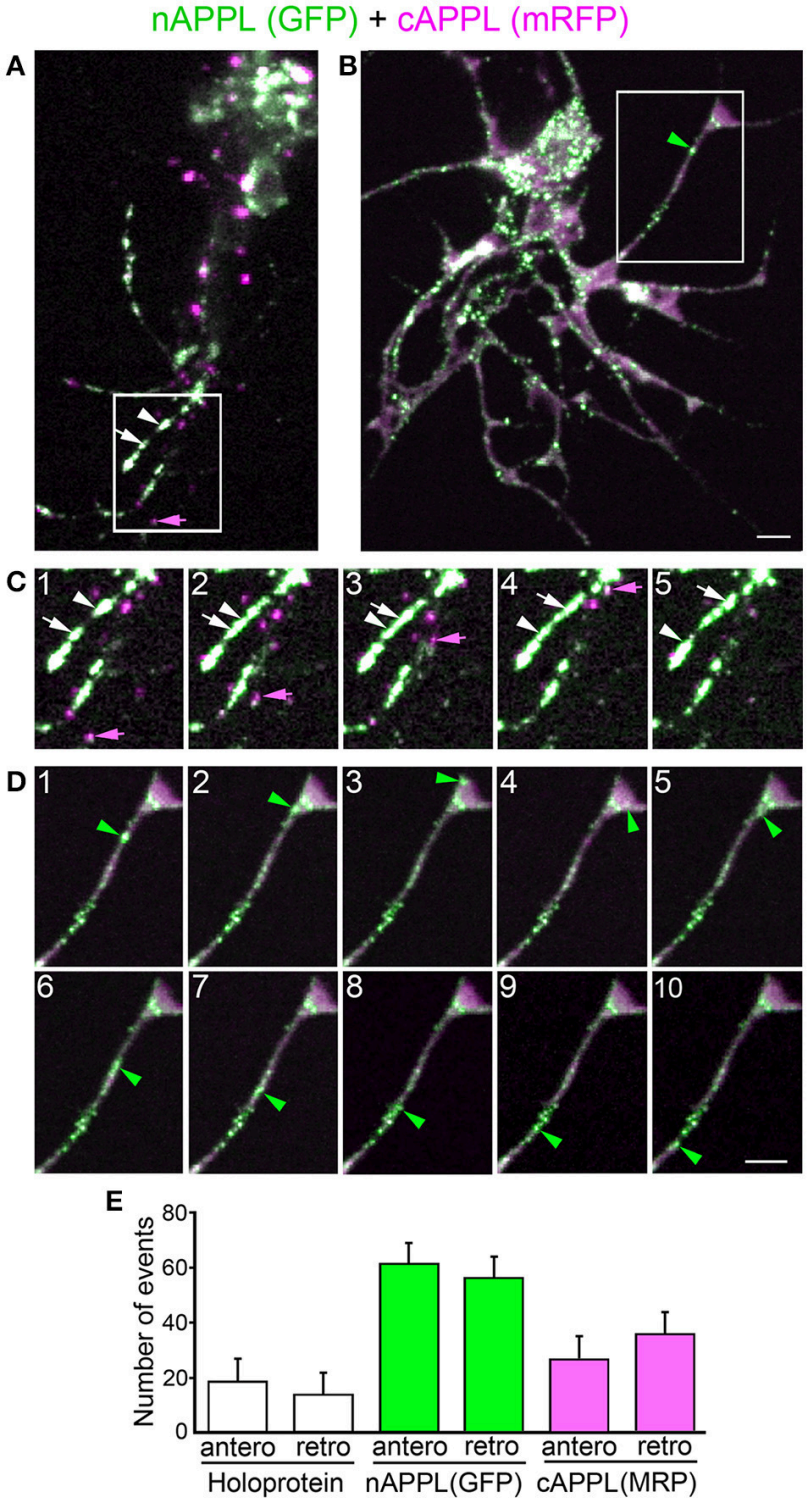
**Dynamic trafficking of fluorescently double-tagged APPL and its fragments in cultured *Drosophila* neurons. (A,B)** Small clusters of partially dispersed *Drosophila* neurons expressing fluorescently double-tagged APPL (via elav-GAL4) at 1 and 4 DIV, respectively. Numerous vesicles containing either N-terminal fragments (green), C-terminal fragments (magenta), or the holoprotein (white immunolabeling) could be seen throughout the neurons and their processes. **(C)** Single frames from a time-lapse of the boxed region in **A** (See also Supplementary Movie S1). White arrow indicates a vesicle containing the holoprotein undergoing retrograde trafficking toward the soma; white arrowhead indicates another vesicle containing the holoprotein in the same neurite undergoing anterograde transport away from the soma. Magenta arrowhead indicates vesicle in a different neurite containing only C-terminal fragments undergoing retrograde transport. **(D)** Single frames from a time-lapse movie of the boxed region in **B** (See also Supplementary Movie S2). Green arrowhead indicates a vesicle containing only N-terminal fragments that was initially transported anterograde to the tip of a growing process (panels 1–5) and then reversed direction (panels 6–10) to undergo retrograde transport back toward the soma (M–Q). Scale bar = 6 μm in **(A,B)**, 2 μm in **(C,D)**. **(E)** Quantification of the number of vesicles containing either APPL holoprotein (white histograms), N-terminal fragments (green histograms), or C-terminal fragments (magenta histograms) that underwent anterograde vs. retrograde transport in time-lapse movies of neurons shown in **(A,B)**. Frames from movies selected at 1 frame/s (approximate transport rates varied from 0.8 to 1.2 μm/s). In **(E)**, each histogram represents average values calculated for 15–20 cells per group, 4 events per cell.

To investigate the dynamic nature of the vesicle populations containing different APP/APPL fragments, we also performed time-lapse imaging of primary neurons expressing our constructs under the control of elav-GAL4. By 1 day in culture (Figure 11A), many of the neurons had extended elaborate processes with a diversity of fluorescently labeled vesicle populations, some of which contained the holoprotein and others that contained either N- or C-terminal fragments. Even after 4 DIV (Figure 11B), neurons with motile processes continued to exhibit a similar complex pattern of APPL trafficking and processing. In most neurons, all three vesicle subtypes underwent bi-directional transport in both proximal and distal domains of the neurons (Supplementary Movies S1, S2). As illustrated in Figure 11C (boxed region in Figure 11A), we observed a substantial number of vesicles containing the holoprotein moving in either anterograde (white arrowhead) or retrograde directions (white arrow); Figure 11C also shows an example of vesicles containing only C-terminal fragments (magenta) undergoing rapid retrograde movement (magenta arrowhead). Figure 11D (boxed region in Figure 11B) shows an example of a vesicle containing only N-terminal fragments (green) that initially was transported anterogradely into the growth cone of a neurite (Figure 11D, panels 1–5), but then underwent a rapid transition and was transported back toward the cell body (panels 6–10). Quantification of these events showed that the anterograde and retrograde transport of the different vesicle classes occurred with approximately equal frequencies (Figure 11E); apparent transport rates varied from 0.8–1.2 μm/s. However, not all cultured CNS neurons showed the same patterns of APPL trafficking and localization. For example, as shown in Supplementary Movie S3, some neurons expressed double-tagged APPL predominantly as a holoprotein that was dynamically distributed throughout their processes, with comparatively little differential trafficking of N- and C-terminal fragments. As described below, we have discovered that different neuronal subtypes also exhibit dramatically different patterns of APP/APPL cleavage within the CNS, revealing a previously unrecognized degree of cell type-specific regulation that affects this complex process.

### APP family proteins undergo cell-specific patterns of trafficking and processing *In vivo*

**Figure 12:**
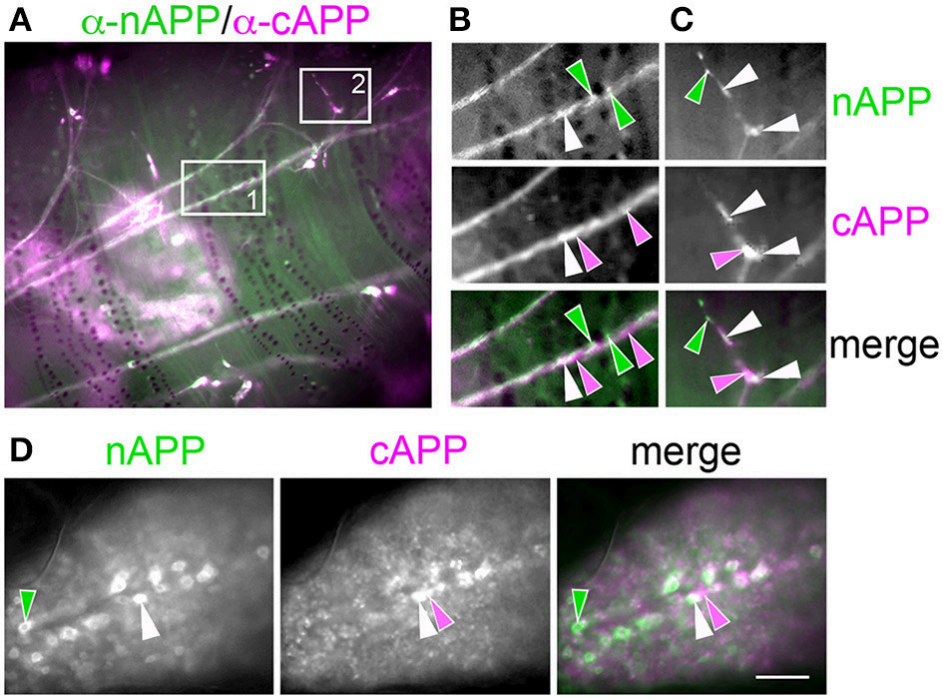
**Double-tagged APP_695_ is trafficked and processed in complex patterns within the larval CNS of *Drosophila***. Images were collected through the cuticle of living 3rd instar *Drosophila* larvae expressing fluorescently double-tagged APP_695_. **(A)** Low-magnification view of abdominal nerves containing motor neurons expressing double-tagged APP_695_ pan-neuronally (via Appl-GAL4). **(B)** Magnified view of boxed region #1 in A. Intermingled transport vesicles containing only N-terminal fragments (green arrowheads), C-terminal fragments (magenta arrowheads), or holoprotein (white arrowheads) could be detected in the fasciculated axons of an abdominal nerve. **(C)** Magnified view of boxed region #2 in A, focused on a neuromuscular junction (NMJ). N-terminal fragments tended to concentrate in the most distal domains of the NMJ (green arrowhead), while C-terminal fragments were more abundant in more proximal domains (magenta arrowhead). Full-length APP (white) was also detectable in certain regions of the NMJ (white arrowheads), reminiscent of the distribution of the holoprotein in some growth cone filopodia but not others (Figure 10). **(D)** Images of neurons within the fused segmental ganglia of the CNS. Different subsets of neurons exhibited markedly different concentrations of N-terminal (green arrowhead) or C-terminal fragments (magenta arrowhead) as well as the holoprotein (white arrowhead). Scale bar = 40 μm in **(A)**; 5 μm in **(B,C)**; 25 μm in **(D)**.

Based on these *in vitro* results, we also examined the patterns of double-tagged APPL and APP_695_ expression at different developmental stages within the *Drosophila* nervous system. When we expressed these constructs pan-neuronally (with Appl-GAL4 *or* elav-GAL4), we could readily visualize the fluorescently tagged proteins in living animals (Figure 12). As with our primary neuronal cultures expressing the double-tagged constructs (Figure 10), nAPPL (GFP), and cAPPL (mRFP) are presented as gray scale images for the individual channels but shown in green and magenta (respectively) in the merged images, whereby co-localization appears white. By focusing through the abdominal cuticle (Figure 12B; magnified box 1 in Figure 12A), we could distinguish transport vesicles containing either N-terminal fragments (green arrowheads) or C-terminal fragments (magenta arrowheads) vs. the holoproteins (white arrowheads) within the motor axons extending to posterior muscles. Within neuromuscular junction regions (NMJ) on the target muscles (Figure 12C; magnified box 2), N-terminal fragments tended to concentrate within the more distal regions of the NMJ, including synaptic boutons (green arrowhead), while C-terminal fragments were more abundant in proximal regions (magenta arrowhead). In contrast, full-length APPL and APP appeared more sparsely distributed throughout the NMJ, only occasionally being detectable within boutons (Figure 12C, white arrowheads). These results support previous evidence that APP family proteins are rapidly processed and removed from synaptic membranes soon after their insertion (Ashley et al., 2005).

**Figure 13:**
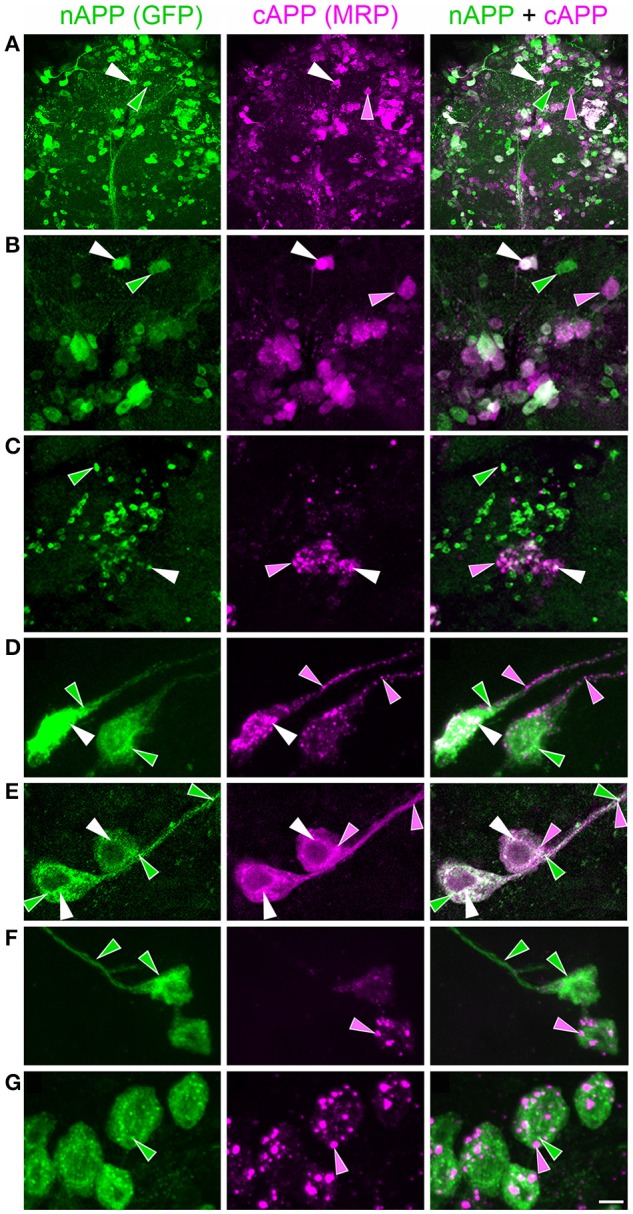
**Different CNS neurons process double-tagged APP_695_ in diverse patterns within the CNS of *Drosophila***. Panels show neurons within the brains of *Drosophila* adults expressing double-tagged APP_695_ under the control of different GAL4 driver lines. **(A)** Neurons in the brain of an adult fly expressing double-tagged APP_695_ pan-neuronally (via Appl-GAL4). Intermingled populations of neurons showed enhanced concentrations of either N-terminal fragments (green), C-terminal fragments (magenta), or the holoprotein (white) in their somata. **(B)** Higher magnification view of the brain in **A** (arrowheads indicate the same neurons at both magnifications). **(C–G)** Expression of double-tagged APP_695_ in dopaminergic neurons (via Ddc-GAL4). **(C)** Low magnification view of the adult brain revealed that different subsets of dopaminergic neurons process APP_695_ in dramatically different patterns, whereby many neurons predominantly accumulated N-terminal fragments in their somata (green); a smaller number predominantly accumulated C-terminal fragments (magenta). White arrowhead indicates larger vesicles containing the holoprotein. **(D)** Example of two dopaminergic neurons that showed robust accumulation of N-terminal fragments within numerous small vesicles in their somata (green arrowheads), as well as larger vesicles containing the holoprotein (white arrowheads). In contrast, C-terminal fragments were concentrated in more sparse vesicle populations in their primary neurites (magenta arrowheads). **(E)** Another pair of dopaminergic neurons that exhibited abundant small vesicles containing C-terminal fragments throughout their somata and primary neurites (magenta), interspersed with more variable vesicle populations containing N-terminal fragments or the holoprotein. **(F)** Two octopaminergic neurons expressing double-tagged APP_695_ (via Tdc-GAL4), exhibiting concentrated N-terminal fragments in their somata (green) and quite variable populations of vesicles with C-terminal fragments (magenta arrowhead). **(G)** A cluster of cholinergic neurons expressing double-tagged APP_695_ (via ChAT-GAL4), exhibiting numerous small vesicles containing N-terminal fragments (green) and sparse populations of larger vesicles containing C-terminal fragments (magenta). Scale bar = 25 μm in **(A,C)**; 15 μm in **(B)**; 2 μm in **(D–G)**.

Unexpectedly, we observed considerable heterogeneity in the pattern of APP/APPL processing within different CNS neurons when we expressed our constructs pan-neuronally (with elav-GAL4 or Appl-GAL4). Within the fused ventral ganglia of third instar larvae, we found that different neuronal subsets showed markedly different concentrations of N- vs. C-terminal fragments (Figure 12D, green and magenta arrowheads). This heterogeneity was even more dramatic in the adult brain (Figures 13A,B), where some neurons were clearly enriched in the holoprotein (white arrows), while other neurons predominantly contained N-terminal (green arrows) or C-terminal fragments (magenta arrows), and these different neuronal populations were intermingled throughout the brain. Of note is that we detected a similar spectrum of expression patterns with both our APP_695_ or APPL constructs, providing further evidence that these proteins are functionally as well as structurally conserved.

To explore whether these cell-specific patterns of APP/APPL processing corresponded to particular neuronal classes, we also expressed our constructs specifically in dopaminergic neurons (with Ddc-GAL4), octopaminergic neurons (with Tdc1-GAL4), or cholinergic neurons (with ChaAT-GAL4). Once again, we discovered a surprising amount of heterogeneity in the patterns of trafficking and processing within each neuronal subtype. For example, different sets of dopaminergic neurons preferentially accumulated either N-terminal APP_695_ or C-terminal fragments in their cell bodies (Figure 13C). At higher magnification, we observed that some of these neurons concentrated N-terminal fragments in their cell bodies and proximal neurites (Figure 13D, green arrowheads), with C-terminal fragments being localized to more sparse vesicle populations (magenta arrowheads). However, other dopaminergic neurons contained abundant C-terminal fragments that were distributed throughout their somata and neurites (Figure 13E, magenta), with more variable vesicle populations containing N-terminal fragments (green arrowheads) or the holoprotein (white arrowheads). Likewise, when we drove expression in octopaminergic neurons (Figure 13F), we detected highly variable distributions of N- vs. C-terminal fragments in neighboring somata. In contrast, when we drove expression in cholinergic neurons (Figure 13G), we found that N-terminal fragments were generally concentrated in their cell bodies, with C-terminal fragments being localized to a discrete population of larger cytoplasmic vesicles (magenta arrowheads).

### Fluorescently tagged APP family proteins exhibit complex patterns of trafficking *In vivo*

**Figure 14:**
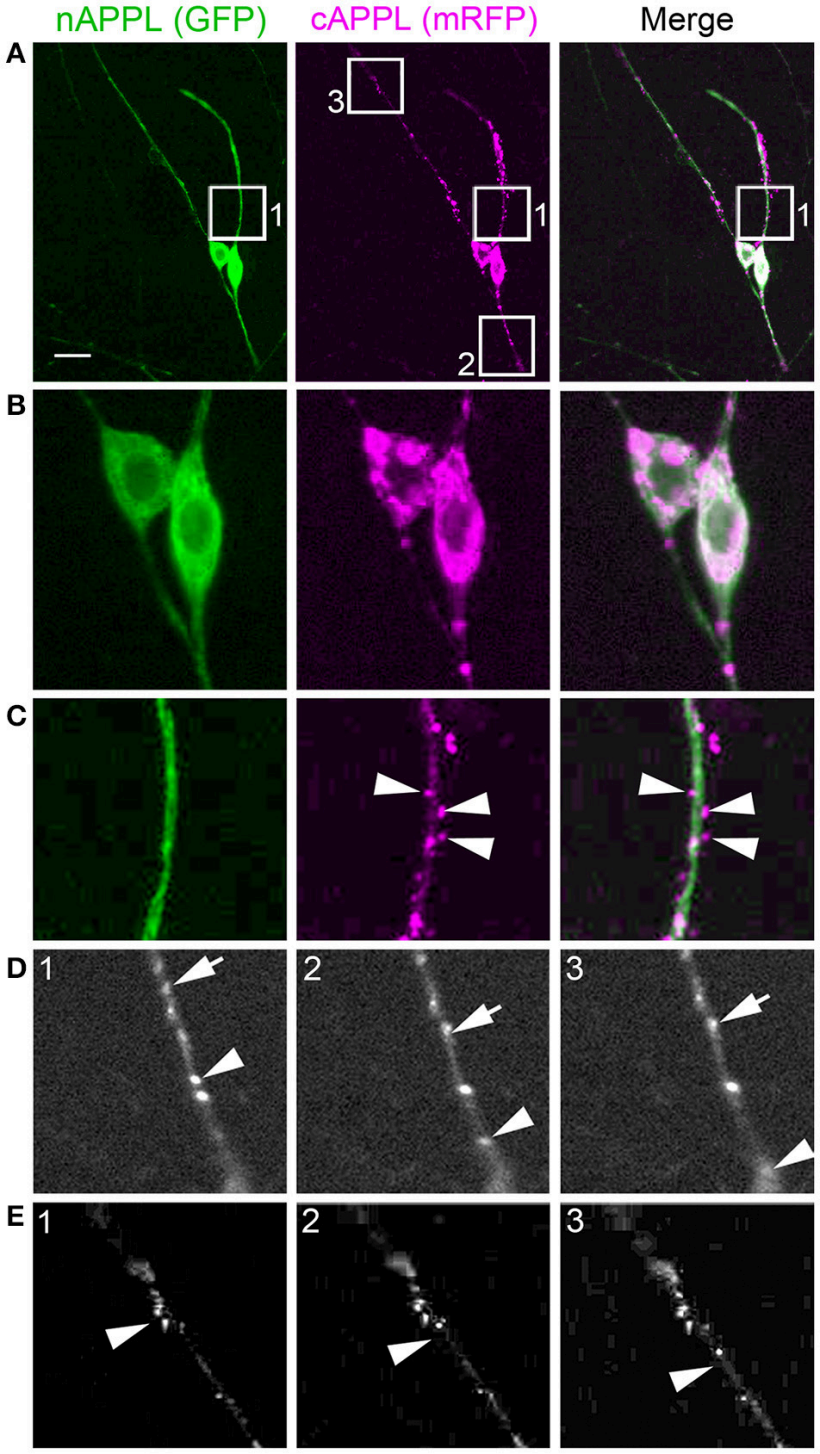
***In vivo* trafficking of fluorescently double-tagged APPL within *Drosophila* CNS neurons**. Two cholinergic neurons expressing double-tagged APPL (via ChAT-GAL4) in the adult fly brain. **(A)** Low magnification view revealed concentrations of the holoprotein (white) in their somata and populations of vesicle containing N-terminal (green) or C-terminal vesicles (magenta) throughout their primary neurites. **(B)** Higher magnification view of the two neurons shown in **(A)**; N-terminal fragments in numerous small vesicles (green) were distributed throughout the somata and primary neurites, while larger vesicles contained C-terminal fragments (magenta) or the holoprotein (white). **(C)** Higher magnification view of boxed region 1 depicted in **(A)** (shown in all three channels). Numerous small vesicles containing N-terminal fragments of APPL were preferentially localized to microtubule tracks within core domain of the primary neurite, while larger vesicles containing C-terminal fragments (arrowheads) were associated with microtubule tracks throughout the circumference of the neurite. **(D)** Single frames from a time-lapse movie taken from box 2 in **(A)** (cAPPL channel only, shown in gray scale); see also Supplementary Movie S4. Arrow and arrowhead indicate two vesicles containing C-terminal fragments undergoing anterograde trafficking (away from the soma). **(E)** Single frames from a time-lapse movie taken from box 3 in **(A)** (cAPPL channel only, shown in gray scale). Arrowhead indicates one of several vesicles containing C-terminal fragments undergoing retrograde trafficking (toward the soma). Scale bar = 8 μm in **(A)**; 2 μm in **(B–E)**. Frames from movies selected every 2 s (approximate transport rates are 1.2 μm/s).

To complement our *in vitro* analysis of APP/APPL dynamics *in vitro*, we also used live cell imaging protocols to visualize double-tagged APPL and APP_695_ in neurons within the CNS. Once again, we found that both holoproteins and their cleavage products were assorted into different vesicle classes that underwent both anterograde and retrograde transport. For example, in cholinergic neurons expressing double-tagged APPL (via ChAT-GAL4; Figure 14), we found that N-terminal fragments (green) localized to numerous small vesicles that were abundant throughout the neurons, while C-terminal fragments (magenta) accumulated in larger vesicles that were distributed more sparsely throughout their neurites (Figures 14A–C). We also noted that vesicles containing either N-terminal or C-terminal fragments appeared to be associated with different sets of microtubules (Figure 14C; boxed region 1): N-terminal APPL fragments (in smaller vesicles) were confined to the core region of neurites, while vesicles containing C-terminal fragments were associated with tracks throughout their entire width. When visualized by time-lapse imaging (Supplementary Movie S4), the smaller vesicles containing N-terminal fragments were difficult to resolve but could be seen moving bi-directionally throughout the processes. Concurrently, larger vesicles containing only C-terminal fragments exhibited cycles of anterograde and retrograde transport throughout the neurites, as shown in time-lapse images from boxed regions 2 and 3, respectively (Figures 14D,E).

**Figure 15:**
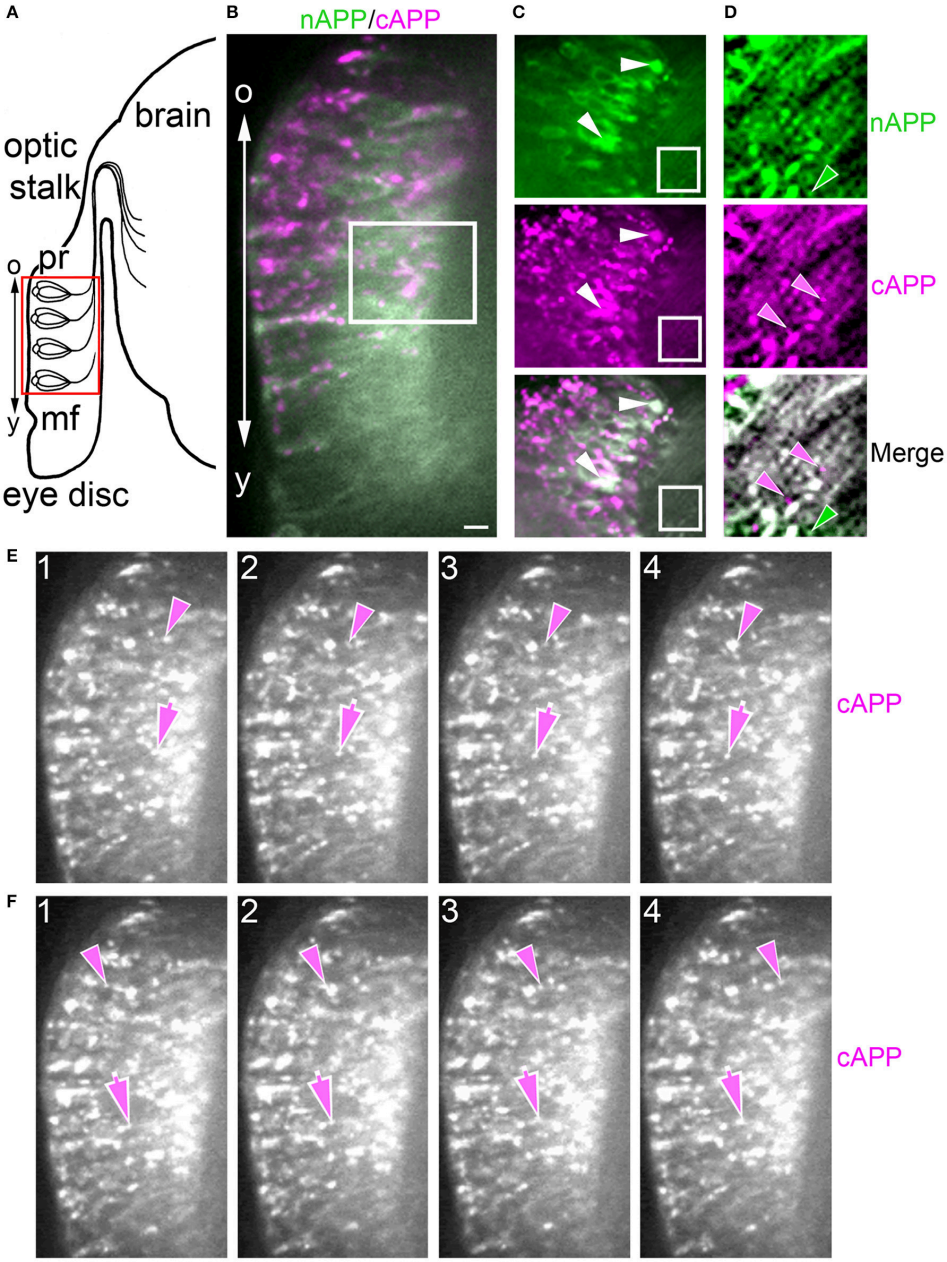
***In vivo* trafficking of fluorescently double-tagged APPL within *Drosophila* photoreceptors is developmentally regulated. (A)** Schematic diagram of the developing eye disc in *Drosophila*; mf = morphogenetic furrow of the eye disc (boundary of photoreceptor differentiation); pr = newly differentiated photoreceptors that are in the process of extending axons through the optic stalk into the optic lobe of the brain; “o” indicates older photoreceptors; “y” indicates younger photoreceptors. Red box indicates the equivalent region of the immunostained preparation shown **(B)**. **(B)** Low magnification view of developing photoreceptors in a cultured eye disc from a larva expressing double-tagged APP_695_ (via GMR-GAL4). Younger photoreceptors (“y”; near the morphogenetic furrow) that had recently begun to extend axons were enriched with vesicles containing the holoprotein (white) and nAPP fragments (green), whereas older photoreceptors (“o”) exhibited many more large vesicles containing cAPP fragments (magenta). **(C)** Higher magnification view of boxed region in **(B)**. N-terminal APP fragments were present in numerous small vesicles throughout the photoreceptors and their axons, while cAPP fragments were concentrated in more dispersed larger vesicles. Photoreceptor somata also exhibited larger cytoplasmic vesicles containing the holoprotein (white arrowheads), similar to other neurons in both *Manduca* and *Drosophila*. **(D)** Higher magnification view of boxed region in **(C)**. Photoreceptor axons contained intermixed populations of vesicles with either nAPP fragments (green arrowhead), cAPP fragments (magenta arrowheads), or the holoprotein (white immunolabeling). **(E,F)** Single frames from a time-lapse of the preparation shown in **(B)** (magenta channel only; see Supplementary Movie S5). Vesicles containing cAPP fragments could be seen moving in both apical **(E)** and basal directions **(F)** within the elongated photoreceptor cell bodies. Scale bar = 5 μm in **(B)**; 3 μm in **(C)**; 0.75 μm in **(D)**; 8 μm in **(E,F)**. Frames from movies selected every 2 s (approximate transport rates are 2 μm/s).

Lastly, to investigate how APP family protein trafficking is regulated in the context of neuronal development *in vivo*, we expressed our constructs specifically in the eye (via GMR-GAL4), and then performed time-lapse imaging of APP/APPL trafficking within developing adult photoreceptors (using cultured larval eye disc-brain preparations). The region of the developing eye disc that we imaged for these experiments is shown schematically in Figure 15A (red box). By taking advantage of the morphogenetic wave of neuronal differentiation that occurs during adult fly eye formation (Ready et al., 1976; Tomlinson and Ready, 1987), we could compare the dynamics of our fluorescently tagged proteins in younger vs. older photoreceptors (Figures 15A,B), coinciding with phases of their initial outgrowth and subsequent synaptogenesis. Intriguingly, we found a marked difference in the relative abundance of the holoprotein vs. its cleavage products in photoreceptors of different ages, once again indicating that APP trafficking and processing is developmentally regulated (similar to our analysis of embryonic neurons). In younger photoreceptors undergoing axon outgrowth (Figure 15B, “y”), we observed a diffuse distribution of the holoprotein (white), while older neurons (“o”) contained more C-terminal fragments (magenta), similar to the accumulation of C-terminal fragments in cultured neurons after they stopped extending processes (Figures 2D, 10D). At intermediate magnification (Figure 15C; boxed region in Figure 15B), we detected a diffuse distribution of N-terminal fragments throughout the photoreceptors (green), while C-terminal fragments were generally confined to larger vesicles (magenta), similar to the distribution of N- and C-terminal fragments seen in other neurons expressing these constructs (Figures 11–14). We also noted the presence of a larger vesicle population containing the holoprotein (Figure 15C, white arrowheads), resembling the amphisome-like vesicles identified in *Manduca* (Figures 5–7). Likewise in regions containing photoreceptor axons growing toward the optic stalk (Figure 15D; boxed region in Figure 15C), we could distinguish different vesicle populations containing either the holoprotein (white immunolabeling), C-terminal fragments (magenta arrowheads), or N-terminal fragments (green arrowheads), recapitulating the differential distribution of APPL cleavage products seen in other developing neurons both *in vitro* and *in vivo*.

When we performed time-lapse imaging of the developing photoreceptors in cultured larval eye disc-brain complexes (Supplementary Movie S5), we also observed distinct vesicles classes containing either N- or C-terminal fragments (or the holoprotein) undergoing complex, dynamic patterns of trafficking. As with the cholinergic neurons shown in Figure 14, we could visualize vesicles containing different fragments moving in both apical (Figure 15E) and basal directions (Figure 15F), although once again, the comparatively small size of vesicle containing N-terminal fragments made them more difficult to resolve. Surprisingly, only ~15% of the vesicles were positive for both GFP and mRFP, indicating that they contain the full-length protein. In addition, we observed twice as many vesicles containing N-terminal fragments compared to vesicles containing C-terminal fragments, which (as noted above) were transported both anterogradely and retrogradely in all developmental stages examined. These *in vivo* results complement our experiments in cultured neurons, providing a novel perspective on the dynamics of APP family proteins in both developing and mature neurons.

## Discussion

Together, our results in both *Manduca* and *Drosophila* provide new evidence that APP family proteins are subject to a complex pattern of trafficking and processing within the nervous system, whereby the intracellular distributions of the holoproteins and their cleavage fragments can be modulated to meet the particular requirements of different neuronal subtypes. As with other APP family proteins, insect APPL is subject to cleavage by a combination of α, β, and γ-secretases (Figures 1G–J), consistent with the identification of all three secretase classes in multiple insect systems (Luo et al., 1990; Ramabhadran et al., 1993; Fossgreen et al., 1998; Greeve et al., 2004; Groth et al., 2010; Bolkan et al., 2012; Prussing et al., 2013; Pace et al., 2014; Bourdet et al., 2015; Lavore et al., 2015; Zheng et al., 2015). Likewise, our studies showed that *Manduca* APPL is preferentially cleaved by α-secretases under normal circumstances, as previously reported for both *Drosophila* APPL and human APP_695_ (Haass et al., 2012; Wentzell et al., 2012; Zhang et al., 2012). We also found that treatment with a combination of α- and γ-secretase inhibitors in our *Manduca* assays resulted in a detectable increase in β-CTF levels (Figure 1H), indicating that blocking α-secretase activity promoted alternative processing by BACE. Similar results have been reported in mammalian systems, whereby diverting APP_695_ from the α-secretase pathway results in enhanced BACE cleavage and elevated Aβ production (Kins et al., 2006; Zhang et al., 2011; Haass et al., 2012). Although the exact residues in APPL that are targeted by secretases have still not been identified, our results support previous evidence that the relative positions of the α- and β-cleavage sites in insect APPL are reversed, relative to their positions in APP_695_ (Carmine-Simmen et al., 2009; Groth et al., 2010). As a result, β-CTFs derived from endogenously expressed APPL are smaller than α-CTFs (Figures 1G,I,J). Nevertheless, recent studies have shown that an Aβ-like fragment derived from *Drosophila* APPL (dAβ) can induce neurodegenerative responses, similar to Aβ fragments derived from APP (Carmine-Simmen et al., 2009; Wentzell and Kretzschmar, 2010). Whether Aβ-like fragments derived from APPL also regulate physiological aspects of neuronal function (as suggested for mammalian Aβ) remains to be explored. Lastly, our detection of C-terminal APPL fragments within neuronal nuclei in both immunolabeled preparations and in neurons expressing the fluorescently tagged constructs (Supplementary Figure S2) supports other evidence that AICD fragments can regulate gene transcriptional responses under both normal and pathological circumstances (Kimberly et al., 2001; Cao and Sudhof, 2004).

By comparing the distributions of endogenous APPL in both *Manduca* and *Drosophila* with epitope-tagged constructs of APPL and APP_695_ (expressed in *Drosophila* neurons), we found that a considerable portion of newly generated APP/APPL is rapidly processed within neuronal cell bodies before being packaged into distinct classes of transport vesicles, so that both N- and C-terminal cleavage products (as well as the holoprotein) can be preferentially targeted to distal sites of release. Similar observations have been reported for APP family proteins in mammalian cells (Back et al., 2007; Muresan et al., 2009; Villegas et al., 2014; Niederst et al., 2015). The fact that we routinely observed all of these vesicle populations undergoing anterograde and retrograde transport (both *in vitro* and *in vivo*) argues that the dynamic distribution of these proteins can be regulated by a combination of intrinsic and extrinsic factors encountered by neurons, depending on their developmental context. Whether newly synthesized APPL must first undergo transcytosis from the plasma membrane (as suggested for APP) or can be directly packaged into secretory vesicles for transport remains to be explored (Kamal et al., 2001; Andersen et al., 2005; Haass et al., 2012; Muresan and Ladescu Muresan, 2015; Niederst et al., 2015). However, our results support the proposal that APP processing can be regulated at multiple sites to match the physiological needs and pathological stresses experienced by neurons within the brain (Kamal et al., 2001; Nikolaev et al., 2009; Szodorai et al., 2009; Niederst et al., 2015).

In both our *in vitro* and *in vivo* assays, we found that APPL trafficking closely matched the motile behaviors of developing neurons, whereby the holoprotein became concentrated in their leading processes and growth cones of their elongating axons (Figures 2, 4, 10). Once inserted into the plasma membrane, however, the holoprotein is rapidly cleared by secretase processing, providing a mechanism for preventing its over-accumulation while terminating APPL-dependent signaling responses. These results are consistent with previous studies showing that APP family proteins are upregulated during periods of active migration and outgrowth in mammalian cells (De Strooper and Annaert, 2000; Sabo et al., 2003; Rice et al., 2012; Sosa et al., 2013), but then undergo rapid turnover once inserted into the plasma membrane (Lyckman et al., 1998; Perez et al., 1999; Tam and Pasternak, 2015; van der Kant and Goldstein, 2015). Similarly, work in *Drosophila* has shown that APPL expression is dynamically regulated during metamorphosis and following acute brain injury, whereby APPL levels are reduced in axons undergoing retraction and dramatically upregulated during periods of rapid regrowth and synaptogenesis (Torroja et al., 1996; Leyssen et al., 2005). How these patterns are modulated in a cell type-specific manner remains to be determined. Nevertheless, our results provide new evidence that the dynamic regulation of APP family proteins in developing neurons is precisely coordinated with their motile behaviors, providing an important mechanism for modulating their responses to cues that affect neuronal differentiation.

In addition, our studies revealed a previously unrecognized aspect of APP/APPL trafficking that is also developmentally regulated. Prior to the onset of EP cell migration (in cultured embryos) or neurite outgrowth (in primary neurons), we found that a substantial proportion of the holoprotein was initially targeted to an intracellular compartment resembling Rab7/Rab11-positive amphisomes (Figures 2, 5, 7), which have been implicated in a variety of signaling pathways as well as autophagosome generation (Patel et al., 2013; Sanchez-Wandelmer and Reggiori, 2013; Bader et al., 2015). Once the neurons commenced active motility, however, APPL concentrations in this amphisome compartment diminished dramatically, concurrent with the increased trafficking of the holoprotein to their leading processes and growth cones (as noted above). Only when neurons had completed their differentiation and established mature synaptic connections did we again observe an increase in amphisomes containing APPL. Most neurons within the developing *Manduca* CNS also contained these amphisome-like vesicles enriched in APPL (Figure 6), as noted in previous studies on *Drosophila* (Torroja et al., 1996). These observations suggest that the transient sequestration of APP family proteins into this compartment might provide a novel developmental mechanism for regulating interactions between the holoproteins (or their cleaved ectodomains) with other membrane-associated signaling complexes involved in neuronal growth, synaptic plasticity, and injury responses (Kogel et al., 2012; Wentzell et al., 2012; Milosch et al., 2014).

With respect to our analysis of APPL processing in insect neurons, it is noteworthy that we obtained virtually identical results by immunolabeling endogenously expressed APPL or by expressing fluorescently tagged APPL (or APP_695_) under the control of the endogenous *Appl* promoter. This congruency gives us increased confidence that we have identified authentic patterns of APP trafficking and processing in healthy neurons, a point that has been contentious in past studies (Muresan and Ladescu Muresan, 2015; Niederst et al., 2015). In addition, we obtained similar patterns of immunolabeling using multiple fixation protocols and antibodies against each domain of APPL (as indicated in Figure 1A), providing further evidence that the subcellular distributions that we reported for different cleavage fragments were not simply the result of epitope masking. In addition, all of our N-terminal APPL antibodies (but not C-terminal antibodies) strongly labeled hemocyte macrophages in *Manduca* that surveil the developing nervous system (Figures 3, 6, “m”). In unpublished studies, we used a variety of methods to show that these macrophages do not themselves express APPL but rather actively phagocytose soluble sAPPL ectodomains both *in vitro* and *in vivo*. Based on other evidence that APP/APPL fragments can induce both neuroprotective and neurotoxic responses (Wentzell et al., 2012; Milosch et al., 2014), we postulate that the sequestration of sAPPL fragments by nearby macrophages may protect the developing nervous system from these bioactive cleavage products. Likewise, we detected a distinct population of smaller cells within the developing CNS of *Manduca* that labeled with anti-nAPPL but not anti-cAPPL (Figure 6, yellow arrows). The position and morphology of these cells suggest that they might represent one of the astrocyte-like glial populations identified in insects (Meyer et al., 1987; Oland et al., 1999; Edwards and Meinertzhagen, 2010; Freeman, 2015), which (like the peripheral macrophages) may scavenge sAPPL fragments released by neighboring neurons. Alternatively, they might comprise a distinct neuronal subtype that preferentially accumulates nAPPL cleavage products within their somata, similar to our observations in *Drosophila* (Figure 13). Additional studies combining glial-specific markers with our sAPPL uptake assays should help resolve this issue.

Another unexpected finding revealed by our experiments using double-tagged APPL and APP_695_ constructs is that different neuronal populations within the CNS processed the holoproteins in remarkably different patterns. When we induced the expression of these constructs with pan-neuronal drivers (Appl-GAL4 or elav-GAL4), we found that some neurons maintained robust levels of the holoprotein, while others preferentially accumulated either N- or C-terminal fragments in their somata (Figure 12). Particularly striking were the diverse patterns of APPL processing that we observed when we expressed the double-tagged proteins in specific subsets of CNS neurons: even within neuronal groups sharing the same transmitter phenotype, we found surprisingly variable distributions of the holoproteins vs. N- and C-terminal fragments in their cell bodies and processes (Figure 13). These observations reveal unexpected complexity in the manner by which different types of neurons regulate the expression and distribution of APP family proteins. These results are also reminiscent of a previous study in *Drosophila*, which revealed the preferential accumulation of different APPL cleavage fragments within neuropil regions targeted by specific neuronal populations (Torroja et al., 1996). We postulate that our results reflect cell-specific differences in the roles that APP family proteins may serve in both the embryonic and mature nervous system (Nicolas and Hassan, 2014; Nhan et al., 2015; van der Kant and Goldstein, 2015).

Lastly, our experiments in the developing ENS of *Manduca* support the model that APP family proteins can function as Goα-coupled receptors under both developmental and pathological conditions (Nishimoto et al., 1993; Okamoto et al., 1996; Niikura et al., 2004). In previous work, we demonstrated that hyperactivation of APPL-Goα signaling induced local retraction and stalling by the migratory EP cells (Ramaker et al., 2013). We also have now shown that this response is normally regulated by *Manduca* Contactin, which is expressed by glial populations that ensheath the EP cells and acts as a functional ligand for APPL (Ramaker et al., 2016). Our current studies have now shown that treating the EP cells with α-secretase inhibitors caused both a marked increase in membrane-associated APPL and a significant inhibition of migration and outgrowth (Figure 8), recapitulating the effects of hyperstimulating APPL or Goα signaling in the developing ENS (Horgan and Copenhaver, 1998; Ramaker et al., 2016). By comparison, neither β- nor γ-secretase inhibitors significantly altered membrane levels of APPL or affected migration, although they did affect other aspects of APPL processing (as described above). Given that α-secretases might target other neuronal receptors that affect the motile behavior of the EP cells (Seals and Courtneidge, 2003; Blobel, 2005; Kuijper et al., 2007), additional experiments will be needed to test whether these effects are specifically due to elevated levels of APPL in their leading processes. Nevertheless, our results suggest that the dynamic regulation of APPL trafficking and processing in the developing insect nervous system plays a critical role in modulating neuronal behaviors mediated by the holoprotein (and possibly its cleavage fragments), providing a new perspective on how APP family proteins might contribute to a wide variety of cell type-specific responses within the embryonic and adult nervous system.

## Author contributions

JR helped design and conduct the experiments using *Manduca* embryos, documented and helped analyze the results, helped prepare the figures, and critically revised the manuscript. RC helped design and conduct the live-imaging experiments using intact *Drosophila* and isolated CNS preparations, documented and helped analyze the results, and provided critical input on the manuscript. TS helped design and conduct most of the biochemical analysis of APPL processing in *Manduca*, documented and helped analyze the results, helped prepare the figures, and critically revised the manuscript. HQ helped design and conduct the experiments using cultured *Drosophila* neurons, documented and helped analyze the results, and contributed to an earlier version of the final manuscript. MC helped design and conduct the biochemical analysis of APPL processing in transgenic *Drosophila*, documented and helped analyze the results, and provided critical input on the manuscript. DK and PC (senior authors who contributed equally to this study) conceived and designed experiments, interpreted the results and supervised statistical analyses of the data, helped generate and edit the figures, and wrote and revised the manuscript (approved by all authors).

## Funding

This work was funded in part by NIH grants NS078363 and AG025525 to PC and NS047633 to DK. In addition, PC and DK also received support from OHSU Presidential Bridge Funding Awards, and DK received support from a grant from the Medical Research Foundation of Oregon. JR and MC were supported in part by grants from the Oregon Partners for Alzheimer's Research; MC was also supported by a grant from the Collins Medical Trust, while JR was also supported by NIA training grant #AG023477. Confocal imaging was supported in part by NIH P30 NS061800 and by the OHSU School of Medicine Faculty Innovation Fund (to PC). The authors declare no competing financial interests.

### Conflict of interest statement

The authors declare that the research was conducted in the absence of any commercial or financial relationships that could be construed as a potential conflict of interest.

## Abbreviations

Aβ: beta-amyloid peptide derived from APP
AICD: APP Intracellular Domain cleavage fragments of APP family proteins
APP: Amyloid Precursor Protein
APP_695_: predominant isoform of APP in mammalian neurons (695 amino acids)
APLP1 and APLP2: APP-Like-Proteins 1 & 2 (additional APP family members expressed in the mammalian brain)
APPL: APP-Like the insect ortholog of human APP
BACE: β-secretase
CTF: C-terminal cleavage fragments of APP family proteins
dAβ: Aβ-like peptide fragment derived from APPL
DIV: Days *in vitro*
en: esophageal nerve of the foregut ENS in *Manduca*
ENS: enteric nervous system
EP cells: neurons within the Enteric Plexus of the *Manduca* ENS
HPF: hours post-fertilization
Kuz: Kuzbanian (ADAM 10 ortholog)
PBS: phosphate-buffered saline
PBST: PBS plus Tween-20
Psn: presenilin
p3: short cleavage fragment generate by α- plus γ-secretase cleavage of APP
sAPP/sAPPL: secreted ectodomain fragments of APP and APPL.
