# Supplementary material for: Amyloid Precursor Proteins Are Dynamically Trafficked and Processed during Neuronal Development
Source: Front Mol Neurosci. 2016 Nov 25;9:130. doi: 10.3389/fnmol.2016.00130 (PMC5122739; doi:10.3389/fnmol.2016.00130)
Supplement: Supplementary file 6 [file DataSheet1.PDF]

Supplementary Material

Amyloid Precursor Proteins Are Dynamically Trafficked and Processed During Neuronal Development

Jenna M. Ramaker, Robert Cargill, Tracy L. Swanson' Hanil Quirindongo, Marlène Cassar, Doris Kretzschmar, and Philip F. Copenhaver *

*** Correspondence:** Dr. Philip F. Copenhaver**:** copenhav@ohsu.edu

Supplementary Figure S1. The large cytoplasmic vesicles containing APPL holoprotein do not express most intracellular compartment markers in GV1 cells. *Manduca* GV1 cells were co-immunostained with a combination of anti-cAPPL and anti-nAPPL antibodies plus antibodies and dyes targeting intracellular organelles (only anti-cAPPL staining is shown in these figures). APPL did not co-localize with most Rab proteins (including Rab 4, 5, 9, and 10), nor with the Golgi marker Lava lamp (lva); the multivesicular body protein VPS4; or the lysosomal marker Lamp1. Likewise, vesicles containing APPL holoprotein did not stain with dyes targeting mitochondria (Mitotracker) or lysosomes (Lysotracker), nor did anti-Rab7 colocalize with Lysotracker (bottom panels). In contrast, as shown in figure 7, both Rab7 and Rab11 colocalized with APPL-containing vesicles. Scale bar = 7 μm.

Supplementary Figure S2. Candidate AICD fragments in the nuclei of cultured *Drosophila* neurons expressing double-tagged APPL. In some preparations, labeling cultured *Drosophila* neurons expressing double-tagged APPL with anti-cAPPL antibodies stained both the large cytoplasmic vesicles containing the holoprotein (arrow) and a variable number of small puncta (arrowheads) within the nucleus (dashed white line). This distribution was most apparent during the first 4 days of growth *in vitro*, which is consistent with the trafficking of APPL intracellular domains (AICDs) into the nucleus to regulate gene transcription. Scale bar = 2 μm.

Supplementary Movie S1. Dynamic trafficking of fluorescently double-tagged APPL and its cleavage products in cultured *Drosophila* neurons. Movie shows a small cluster of larval CNS neurons expressing double-tagged APPL (via *elav-GAL4*) at day 1 after plating. Distinct vesicle

populations containing either N-terminal fragments (green), C-terminal fragments (red), or the holoprotein (white) are intermingled throughout the neurons and their processes and exhibit both anterograde and retrograde trafficking. Selected frames from this movie are shown in Figure 11C.

Supplementary Movie S2. Dynamic trafficking of fluorescently double-tagged APPL and its cleavage products continue in older cultured *Drosophila* neurons. Movie shows a similar cluster of larval CNS neurons expressing double-tagged APPL (via *elav-GAL4*) after 8 DIV. As in Supplementary Movie S1, different vesicle populations containing N-terminal fragments (green), C-terminal fragments (red), or the holoprotein (white) exhibit both anterograde and retrograde trafficking. Selected frames from this movie are shown in Figure 11D.

Supplementary Movie S3. Dynamic trafficking of fluorescently double-tagged APPL holoprotein in older cultured *Drosophila* neurons. Movie shows another small cluster of larval CNS neurons expressing double-tagged APPL (via *elav-GAL4*) after 8 DIV. In contrast to the neurons shown in Supplementary Movies S1-S2, the vast majority of double-tagged APPL is distributed throughout the neurons as a holoprotein (shown in yellow). Vesicles containing the holoprotein can be seen moving in both anterograde and retrograde directions.

Supplementary Movie S4. Dynamic trafficking of fluorescently double-tagged APP_{695} and it cleavage products in cholinergic neurons *in vivo*. Movie shows a pair of cholinergic neurons expressing double-tagged APP_{695} (via *ChAT-GAL4*) in the adult fly brain. Panel 1: N-terminal fragments (green in panel 3) were localized within numerous small vesicles that could be seen moving bi-directionally but were difficult to resolve individually. Panel 2: C-terminal fragments (red in panel 3) occupied larger, more sparsely distributed vesicles that underwent both anterograde and retrograde transport. Selected frames from this movie are shown in Figure 14D-E.

Supplementary Movie S5. Dynamic trafficking of APP in developing photoreceptors. Movie shows photoreceptors within the developing eye disk of a late 3rd instar larva, expressing double-tagged APP_{695} (via *GMR-GAL4*). Older photoreceptors (distal from the morphogenetic furrow) are toward the top; younger, more recently differentiated photoreceptors are towards the bottom. Left panel shows GFP (N-terminal APP_{695}). Middle panel shows mRFP (C-terminal APP_{695}). Right panel shows merged movie of both channels. N-terminal fragments (green) were localized within numerous small vesicles that could be seen moving bi-directionally but were difficult to resolve individually. C-terminal fragments occupied larger, more sparsely distributed vesicles that underwent both anterograde and retrograde transport. Younger photoreceptors also were more enriched with vesicles containing the holoprotein (white) and N-terminal fragments (green), whereas older photoreceptors accumulated substantially more C-terminal fragments in more prominent vesicles within their cell bodies and axons. Selected frames from this movie are shown in Figure 15D-E.
