# Supplementary figures and images for: Amyloid Precursor Proteins Are Dynamically Trafficked and Processed during Neuronal Development
Source: Front Mol Neurosci. 2016 Nov 25;9:130. doi: 10.3389/fnmol.2016.00130 (PMC5122739; doi:10.3389/fnmol.2016.00130)

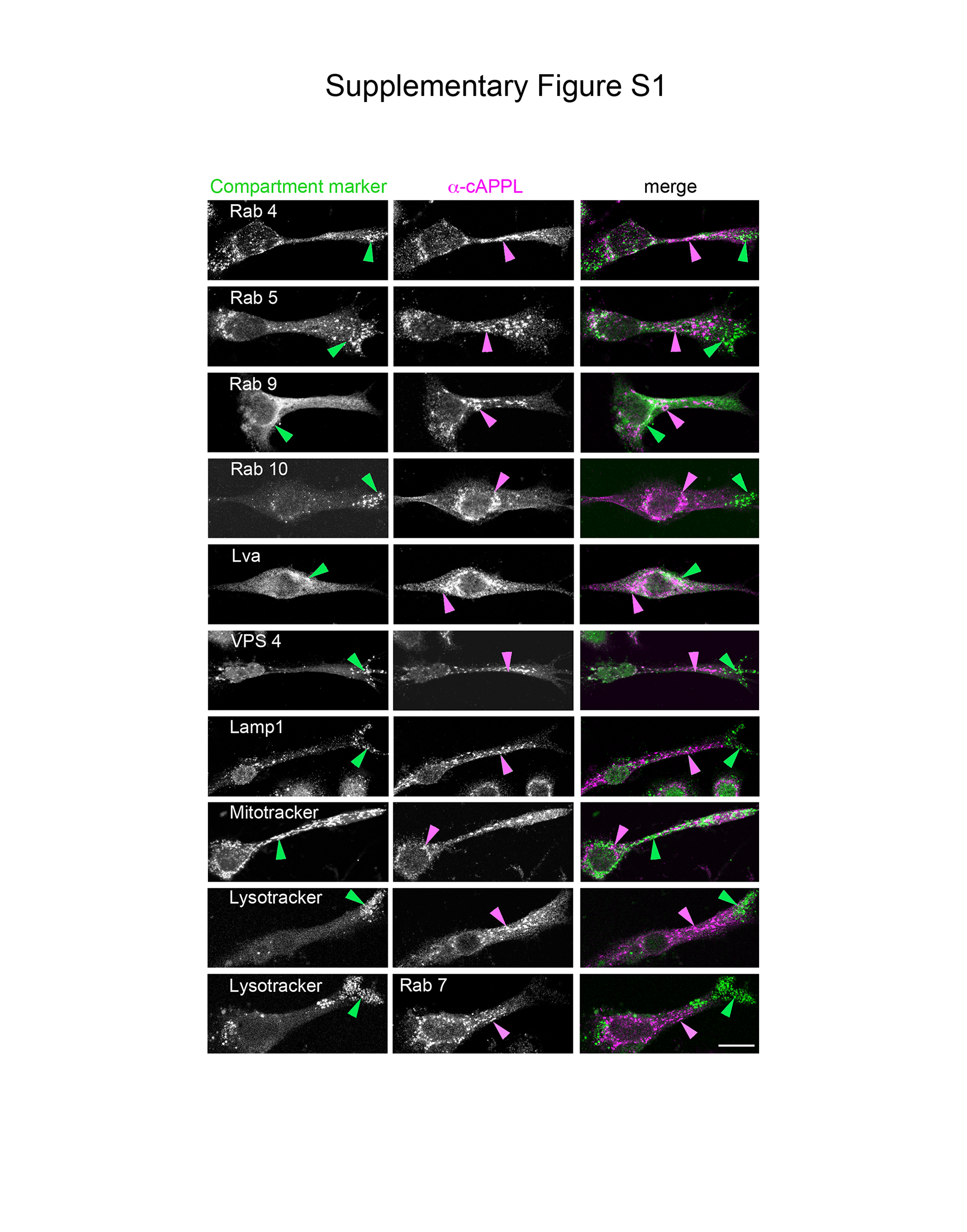

Supplement: Supplementary file 7 [file Image1.TIFF]

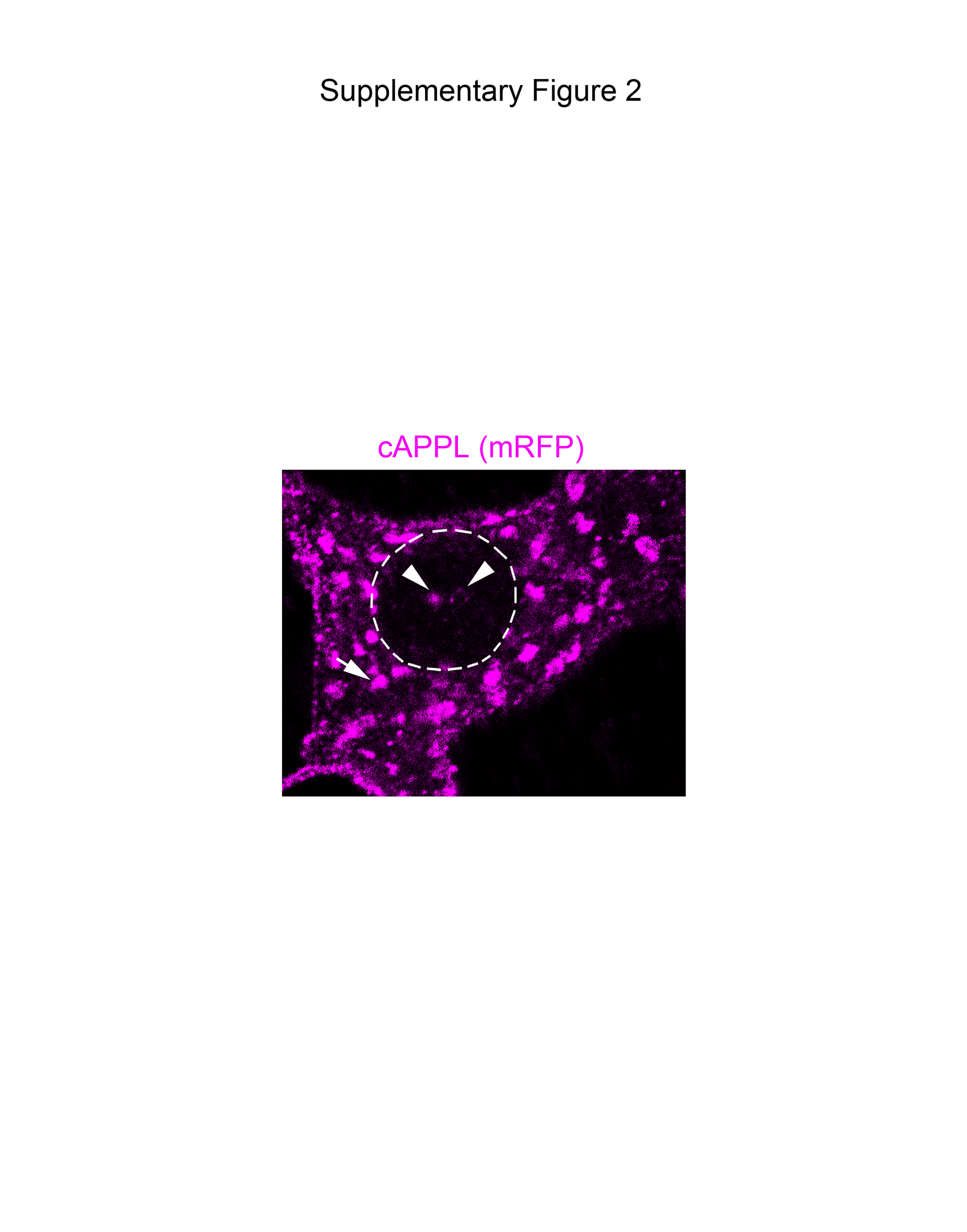

Supplement: Supplementary file 8 [file Image2.TIFF]
